# A quantitative Lewy-fold-specific alpha-synuclein seed amplification assay as a progression marker for Parkinson’s disease

**DOI:** 10.1007/s00401-025-02853-y

**Published:** 2025-02-20

**Authors:** Alexander M. Bernhardt, Sebastian Longen, Svenja V. Trossbach, Marcello Rossi, Daniel Weckbecker, Felix Schmidt, Alexander Jäck, Sabrina Katzdobler, Urban M. Fietzek, Endy Weidinger, Carla Palleis, Viktoria Ruf, Simone Baiardi, Piero Parchi, Günter U. Höglinger, Torsten Matthias, Johannes Levin, Armin Giese

**Affiliations:** 1 Department of Neurology, Ludwig-Maximilians-Universität München, Munich, Germany; 2 Aesku.Diagnostics GmbH, Wendelsheim, Germany; 3 MODAG GmbH, Wendelsheim, Germany; 4 Department of Biomedical and Neuromotor Sciences (DiBiNeM), University of Bologna, Bologna, Italy; 5 Munich Cluster for Systems Neurology (SyNergy), Munich, Germany; 6 Center for Neuropathology and Prion Research, Faculty of Medicine, Ludwig-Maximilians-University Munich, Munich, Germany; 7 IRCCS, Istituto Delle Scienze Neurologiche Di Bologna, Bologna, Italy

## Abstract

Misfolded α-synuclein (αSyn) is the hallmark of α-synucleinopathies such as Parkinson’s disease (PD), dementia with Lewy bodies (DLB), and multiple system atrophy (MSA). While seed amplification assays (SAA) have demonstrated ultrasensitive detection of misfolded αSyn, they have been primarily used reliably to provide binary (positive/negative) results for diagnostic purposes. We developed an SAA with enhanced specificity for Lewy-fold α-synucleinopathies and introduced a quantifiable measure correlating with clinical severity. Cerebrospinal fluid (CSF) of 170 patients with neurodegenerative diseases and controls was analyzed. Blinded measurements demonstrated 97.8% sensitivity and 100% specificity for Lewy-fold α-synucleinopathies, correctly identifying PD and DLB while excluding MSA. In addition, we validated the strain specificity of the assay by testing brain homogenates from 30 neuropathologically confirmed cases. A novel Lewy-fold pathology (LFP) score based on positive signals in a dilution series provided a quantitative measure of αSyn seeds. The LFP score significantly correlated with motor and cognitive impairment presented by Hoehn and Yahr stage, MDS-UPDRS III, and MoCA. Longitudinal tracking in seven PD cases showed progressive LFP score increases corresponding with clinical deterioration, highlighting the assay’s potential for monitoring disease progression at an individual level. Our Lewy-fold-specific SAA enhances ante-mortem diagnosis and differentiates Lewy-fold α-synucleinopathies from MSA. Unlike previous assays, the LFP score offers a quantitative assessment, showing promise as a progression marker and pharmacodynamic biomarker for αSyn-targeting therapies. This represents an important step toward developing an αSyn SAA that could help to track disease progression quantitatively, with potential applications in both clinical diagnostics and therapeutic trials.

**Supplementary Information:**

The online version contains supplementary material available at 10.1007/s00401-025-02853-y.

## Introduction

Alpha-synucleinopathies, a common group of neurodegenerative diseases, are distinguished by pathological deposits of the α-Synuclein (αSyn) protein. Parkinson’s disease (PD), the most prominent α-synucleinopathy, features Lewy bodies and Lewy neurites—intracellular inclusions containing misfolded αSyn—resulting in neuronal loss, motor and cognitive impairments. Dementia with Lewy bodies (DLB) is diagnosed when cognitive decline precedes motor symptoms, both conditions being neuropathologically classified as Lewy body disease (LBD). Unlike multiple system atrophy (MSA), a rapidly progressive and fatal α-synucleinopathy with no effective therapy [56], PD progresses more slowly, and patients benefit from symptomatic therapies such as pharmacotherapy or deep brain stimulation. Diagnostic criteria for these diseases include physical and neurological examinations as well as neuroimaging tests [32, 38]. Differentiating between PD and MSA remains challenging due to the lack of approved objective diagnostic tests targeting αSyn pathology.

Recent advancements in biomarker development have refined Seed Amplification Assays (SAA) to detect small quantities of pathological αSyn seeds in cerebrospinal fluid (CSF) of patients with α-synucleinopathies. Initially developed for prion diseases [2, 3, 45], SAAs are now considered promising tools for early and accurate diagnosis. A clear distinction between MSA and PD using SAAs is still challenging but highly needed in clinical practice. Different conformations of fibrillar αSyn in MSA and PD/DLB have been identified: two MSA-fold strains in MSA [46] and one Lewy-fold strain in PD and DLB [60]. This necessitates an αSyn SAA with enhanced specificity for detecting Lewy-fold seeds. Current αSyn SAAs show promise [39, 41] with a recent meta-analysis revealing high diagnostic sensitivity and specificity for PD and DLB [61], though sensitivity for MSA is lower. Some studies suggest that αSyn SAAs can differentiate PD/DLB from MSA through distinct kinetic profiles [47, 48, 51], though sensitivity for MSA is only 80% in these publications. Notably, CSF αSyn SAA positivity is included as a supportive biomarker in the MDS criteria for diagnosing MSA [57] and a key feature in proposed biological definitions of PD [21, 50]. Emerging disease-modifying strategies for α-synucleinopathies [34, 54] underscore the need for precise diagnostic tools.

There is a critical need for reliable progression markers in α-synucleinopathies. Although SAAs exhibit high specificity, developing a quantifiable SAA that accurately correlates with clinical severity and disease progression is challenging. Attempts have focused on kinetic parameters of the SAA reaction or obtaining an SD_50_ (50% seeding dose) through serial dilutions. Despite evidence of a connection between kinetic profiles and seed concentration in prion protein SAAs, the relationship is more complex for endogenous patient-derived seeds in a biological matrix, given variability between technical replicates. Proposed quantitative readouts for kinetic profiles depend on the SAA experimental setup [44] and include several kinetic measures [8, 9] that show some correlations with cognitive and motor impairment. However, a joint study by three independent labs [44] showed that while diagnostic reproducibility is high, the SD_50_ correlated only with disease duration, but not motor or cognitive impairments. Brain homogenate and CSF studies argue in favor of dilution series as a measure of seed concentration for developing a quantifiable αSyn SAA [5, 31].

Quantifiable αSyn SAAs, once approved for clinical use, will revolutionize the identification of at-risk patients and enhance recruitment for clinical trials targeting disease prevention. These assays may also serve as disease monitors and pharmacodynamic biomarkers for novel anti-aggregative therapies, such as emrusolmin/anle138b [29, 30, 55], aiding in the evaluation of therapeutic efficacy in clinical trials. Our study aims to address this by introducing a quantifiable measure, the Lewy-fold pathology (LFP) score to an αSyn SAA.

## Methods

### Clinical assessments

Eligible participants were recruited between 2020 and 2023 at the Neurology Department of Ludwig-Maximilians-University (LMU) Munich Hospital. All subjects enrolled in a research protocol with annual reassessments and had at least one follow-up assessment after their baseline visit. When available, neuroimaging results (MRI and FDG, tau PET scan, or DaTscan) were reviewed. All patients provided written informed consent for clinical assessment and lumbar puncture (LP) to collect CSF, with the study approved by the LMU Munich ethics committee (project number 23-0602). Procedures adhered to the 1964 Helsinki Declaration and its amendments.

Patients met MDS diagnostic criteria for the clinical diagnosis of Parkinson’s disease (PD, *n* = 43) [38] and possible or probable dementia with Lewy bodies (DLB, *n* = 3) according to McKeith criteria [32], consensus criteria for probable multiple system atrophy (MSA, *n* = 31) [19] as well as MDS diagnostic criteria for clinically established or probable MSA [57], or possible or probable progressive supranuclear palsy (PSP, *n* = 32) [22]. The diagnosis of 35 patients with Alzheimer’s disease (AD) was made according to the International Working Group criteria [15]. Patients with primary progressive aphasia and behavioral variant frontotemporal dementia (FTD) were diagnosed based on international criteria [20, 40]. Controls had no history of major neurological or psychiatric illness and performed normal on cognition and neurological examination. All patients were examined by board-certified neurologists who collected clinical and demographic data. Disease severity was rated using validated scales (Movement Disorder Society Unified Parkinson's Disease Rating Scale (MDS-UPDRS), Unified Multiple System Atrophy Rating Scale (UMSARS) and Hoehn and Yahr (H&Y) staging and cognitive performance was assessed using the Montreal Cognitive Assessment (MoCA).

### Neuropathological studies

Fresh–frozen human post-mortem brain tissue was obtained from the Neurobiobank of the LMU Munich, including clinically well-documented and neuropathologically confirmed cases of PD (*n* = 10), PSP (*n* = 4), MSA (*n* = 10), and controls (*n* = 6). Donors provided consent for research use of their brains, approved by the research ethics committee of the LMU Munich (no. 345-13).

The Gilman and MDS diagnostic criteria were used to define MSA-related neuropathological changes [19]. The severity of Lewy body pathology was classified according to Braak [7] criteria. Alzheimer pathology was classified using Braak and Braak [6], Thal [53] and NIA [23] classification systems. Comprehensive neuropathological reports were available for all cases, with clinical data gathered from medical records. Two types of tissue were included—cerebellum and frontobasal cortex—to reflect differential affection of brain regions by αSyn pathology in MSA and PD, respectively, as well as availability at the Neurobiobank Munich.

### Brain homogenate preparations

Brain homogenates (BH; 10% w/v) were prepared by homogenizing tissue in PBS supplemented with 1 × protease inhibitor (cOmplete^™^ Protease Inhibitor Cocktail, Roche Diagnostics, Germany) and 1 × phosphatase inhibitor (PhosSTOP^™^, Roche Diagnostics, Germany) using a tissue lyser (Precellys^®^ Evolution, Bertin technologies, France) for 1 min at 6500 rpm. The homogenate was precleared by centrifugation at 10,000 × *g* for 10 min at room temperature and the supernatant was transferred to a new tube and stored at − 80 °C for αSyn SAA analysis. For αSyn SAA testing, BHs were serially diluted in PBS and the 10^–4^ dilutions were used for the SAA.

### Lumbar puncture and CSF handling

After discarding the first 2–3 drops, 10 ml of CSF was withdrawn and collected in 15 ml polypropylene tubes (Sarstedt, Germany). A sample was sent for routine analysis (cell count, total protein, glucose, etc.). The remaining CSF was centrifuged at 2,000 × *g* for 10 min at room temperature, transferred to 0.5 ml tubes (Azenta Life Sciences, Germany) and frozen at − 80 °C within 30 min. The following CSF biomarkers were analyzed with the ELISA Innotest Kit (Fujirebio Europe N.V., Belgium): Aβ_42_, Aβ_40_, Aβ_42/40_ ratio, total-tau and p-tau_181_. The normal cutoff values were Aβ_42_ > 375 pg/ml, Aβ_42/40_ ratio > 5.5%, total-tau < 445 pg/ml and p-tau_181_ < 61 pg/ml.

### Expression and purification of αSyn for generation of in-vitro formed fibrils

Expression of wild-type αSyn was achieved by overnight incubation of BL21(DE) pLysS cells carrying a pET5a-SNCA plasmid [43] in auto-induction medium. The next day, cells were harvested by centrifugation and subjected to osmotic lysis. The lysate was subjected to acidification to pH 3.5, centrifugation and subsequent neutralization. Afterwards, the protein was purified by anion exchange and size exclusion chromatography in PBS. Protein concentration was measured, adjusted to 3 mg/ml and further used for generation of in-vitro formed fibrils.

### Preparation of in-vitro formed fibrils

Artificial αSyn fibrils were generated as described previously [35, 37]. In brief, 3 mg/ml monomeric WT αSyn was incubated in PBS including 0.02% NaN_3_ at 37 °C for 120 h under vigorous shaking (1000 rpm) using an Eppendorf Thermomixer C (Eppendorf, Germany). Subsequently, the fibrils were subjected to sonication using a cup horn sonicator (Q700, QSonica, USA) and the quality of the fibrils was confirmed by Thioflavin T (ThT) fluorescence (T3516, Sigma Aldrich, Germany), sedimentation assay via ultracentrifugation at 100,000 × *g* [35] and transmission electron micrographs (Supplementary Figure S1). Fibrils were stored at − 80 °C.

### Alpha-synuclein seed amplification assay (αSyn SAA)

Monomeric, N-terminally His_6_-tagged αSyn (6,666,637, AIDA GmbH, Germany) was purified in a proprietary process based on standard affinity and anion exchange chromatography methods. After purification, the protein was lyophilized and stored at -80 °C until further use. The αSyn SAA assays were conducted in black 96-well plates with clear bottom (Nunc MicroWell 96-Well Plates, 265,301, Thermo Scientific, USA), each preloaded with six glass beads (A554.1, Carl Roth, Germany) using a bead dispenser (MolGen BV, Netherlands). Each reaction was carried out in a total volume of 100 µl containing 0.07 mg/ml recombinant His_6_-αSyn, 8 µM ThT, reaction buffer and 15% CSF or 2% of a 10^–4^ BH dilution, respectively. A 4 × stock solution of SAA buffer (783,337, AIDA GmbH, Germany) contained 600 mM NaCl, 140 mM phosphate buffer pH 8.0 and 1% of a proprietary detergent solution. Plates were sealed with QuickSeal Micro foil (G070-N, Kisker Biotech, Germany) and subjected to repetitive shaking and rest cycles of 1 min at 42 °C in a fluorescence reader (FLUOstar Omega, BMG Labtech, Germany). ThT fluorescence was measured every hour at an excitation of 448 nm and an emission of 482 nm for a total of 144 h.

**Fig. 1:**
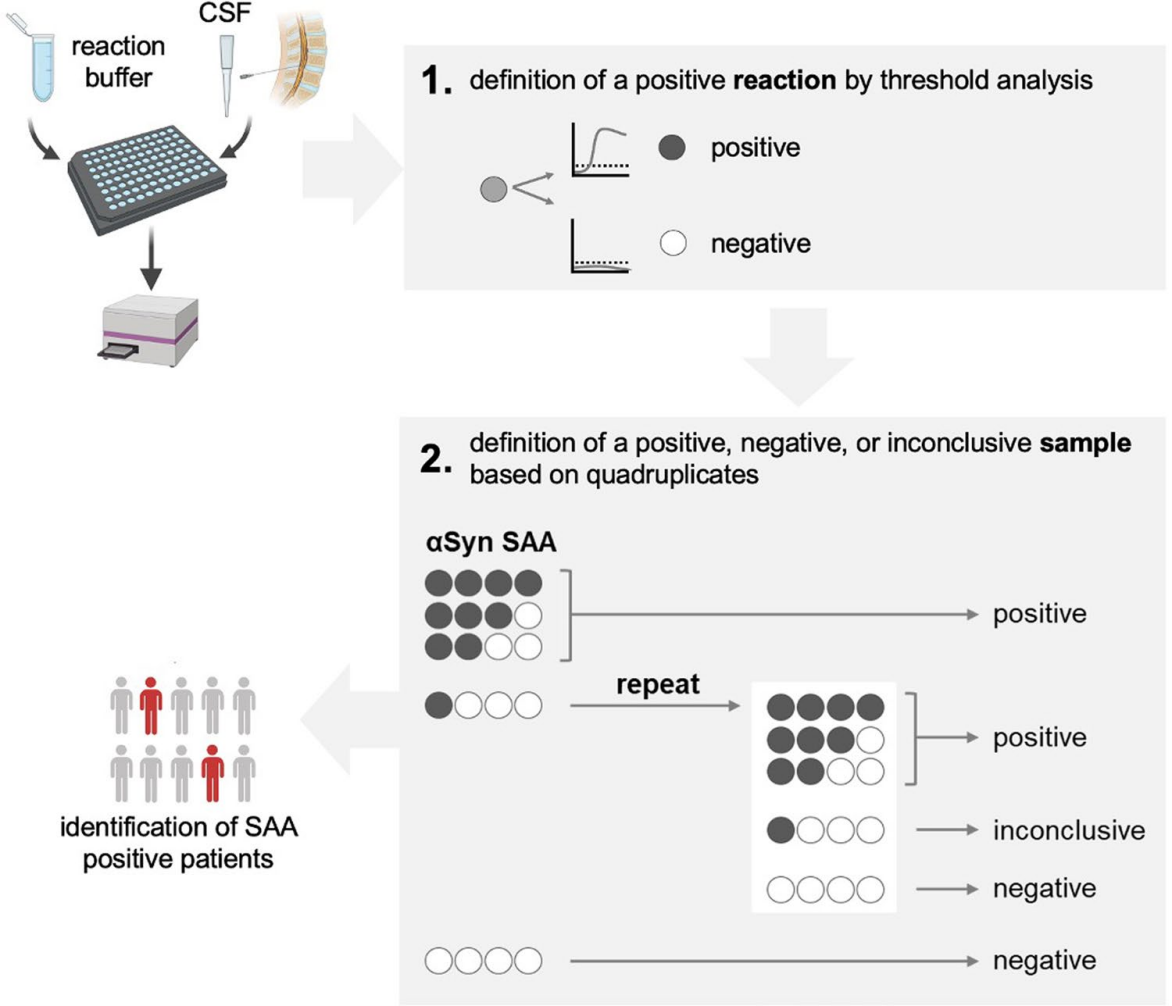
SAA experimental workflow and interpretation of results. For the cohort screening, CSF samples are added to reaction buffer and the experiment is performed in quadruplicates. The analysis involved (1) the definition of a positive or negative single reaction (1, upper grey box) and (2) the definition of a positive, negative, or inconclusive sample based on quadruplicates (2, lower grey box): an individual reaction was considered positive if the fluorescence intensity reached ≥ 15% of the average F_max_ of the positive control (dotted line). The sample was defined positive if at least 2 replicates out of 4 were positive and negative if none of the 4 replicates reached the threshold. 1 out of 4 was considered inconclusive and the sample measurement was repeated. If the result was still 1 out of 4, the sample was declared inconclusive. Partially created with BioRender.com

All samples were measured in quadruplicates. Each plate contained quadruplicates of negatively tested CSF as negative controls and positively tested CSF of PD patients or artificial fibrils spiked in negatively tested CSF (final concentration 7.5 pM) as positive controls. The minimum value for each well was subtracted for normalization. An individual well signal was considered positive if the fluorescence intensity of a reaction reached ≥ 15% of the average maximum fluorescent intensity of the positive control. A patient sample was positive if at least 2 replicates showed a positive signal. Samples with no signal were defined as negative. Samples with 1 of 4 replicates showing a positive signal were considered inconclusive and repeated once. If the result remained 1 of 4, the sample was declared inconclusive (Fig. 1).

### Dilution series and Lewy-fold pathology (LFP) score

All samples tested positive in the initial SAA were subjected to a dilution series and subsequent SAA. For this, samples were diluted (1:2, 1:4, 1:8 and 1:3, 1:10, 1:30, 1:100) using pooled negative control CSF, which had previously been confirmed as devoid of pathological αSyn seeds. The use of pooled negative CSF ensures consistency regarding matrix effects across samples and provides optimal comparability in the dilution series. Dilutions were measured in quadruplicate. Per sample, all positive signals of the dilution series were summed up and defined as the LFP score.

### Statistical analysis

Statistical analyses were performed in R [14], version 4.1.1 and Python [42], version 3.9.18. Numeric demographic and clinical variables were analyzed using Wilcoxon or Student’s *t*-tests after checking for normality (Shapiro–Wilk test). Significance levels were set to *p* < 0.05. All tests were two-sided. Figures were produced using the ggplot2 package (version 3.4.2) [58] and Biorender (Biorender.com). Bivariate correlations of continuous variables with the LFP score were calculated using Pearson correlation coefficients and Spearman correlation coefficients were used for ordinal variables. Error bars indicate the standard deviation (SD), unless otherwise indicated.

## Results

### Clinical characteristics

The study cohort included individuals diagnosed with PD, DLB, MSA, PSP, AD, FTD and controls (CON). MSA patients showed greater motor impairment than PD patients, reflected by higher MDS-UPDRS III scores (46.04 ± 17.87 vs 25.74 ± 11.90) and H&Y stage values (3.00 ± 1.00 vs 2.00 ± 0.50). PSP patients also exhibited higher MDS-UPDRS III scores (43.96 ± 13.85) and H&Y stage values (3.00 ± 1.00) compared to the PD group. These differences underscore the distinct clinical presentations and severities associated with each neurodegenerative condition. In AD cases, ATN biomarkers indicated significant amyloid pathology with low CSF Aβ_42_ levels (604.03 ± 208.07 pg/ml) and a low Aβ_42/40_ ratio (4.29 ± 1.06%), alongside elevated phospho-tau_181_ (84.34 ± 27.36 pg/ml). The clinical and demographic characteristics of the study cohort are summarized in Table 1.

**Table 1:** Clinical characteristics of the study cohort at baseline

Diagnosis		PD	DLB	CON	MSA	PSP	AD	FTD
Age at onset [years]		56.89 ± 10.42	59.33 ± 19.14	NA	57.26 ± 7.79	67.91 ± 7.98	69.29 ± 7.51	67.71 ± 9.30
Disease duration [years]		3.73 ± 2.21	5.33 ± 3.51	NA	4.16 ± 2.40	3.31 ± 1.97	3.69 ± 2.61	3.00 ± 1.73
Sex	male	31	3	6	22	18	14	2
	female	12	0	14	9	14	21	5
UPDRS III at LP		25.74 ± 11.90	38.1 ± 11.9	NA	46.04 ± 17.87	43.96 ± 13.85	NA	NA
Hoehn and Yahr at LP		2.00 (0.50)	2.00 (0.50)	NA	3.00 (1.00)	3.00 (1.00)	NA	NA
CSF Aβ_1-42_ [pg/ml]		1086.21 ± 315.52	786.72 ± 366.61	1215.42 ± 465.72	895.96 ± 306.34	965.15 ± 354.42	604.03 ± 208.07	1156.68 ± 546.56
CSF Aβ_1-40_ [pg/ml]		13523.97 ± 3825.34	10091.67 ± 2361.71	15591.00 ± 5842.06	10593.96 ± 3444.03	12238.79 ± 4416.38	14667.40 ± 5383.42	13468.14 ± 5907.70
CSF Aβ_42/40_ ratio [%]		8.18 ± 1.79	8.20 ± 4.23	8.04 ± 1.71	8.75 ± 1.63	8.15 ± 2.08	4.29 ± 1.06	8.53 ± 1.49
CSF total-tau [pg/ml]		164.47 ± 82.44	144.11 ± 63.20	223.12 ± 109.26	217.08 ± 126.48	237.14 ± 154.92	467.58 ± 231.39	256.98 ± 137.46
CSF phospho-tau_181_ [pg/ml]		44.39 ± 14.42	49.82 ± 31.05	43.45 ± 11.52	40.41 ± 11.56	49.37 ± 20.16	84.34 ± 27.36	49.67 ± 12.70

LP: lumbar puncture, PD: Parkinson’s disease, DLB: dementia with Lewy bodies, CON: control, MSA: multiple system atrophy, PSP: progressive supranuclear palsy, AD: Alzheimer’s disease, FTD: frontotemporal dementia, CSF: cerebrospinal fluid, NA: not assessed

For most clinical features, mean and standard deviation are shown. For H&Y scores, median and interquartile ranges are shown

### CSF αSyn SAA test results

To ensure unbiased evaluation, all CSF samples were measured using our αSyn SAA under blinded conditions. The assay demonstrated high sensitivity for detecting Lewy-fold α-synucleinopathies (97.8%, PD and DLB) and 100% specificity in distinguishing Lewy-fold α-synucleinopathies (PD and DLB) from MSA and CON (Table 2). Among CSF samples from PD/DLB, 42 PD and 3 DLB tested positive, while none of the MSA or CON CSF samples were positive. One PD CSF sample was considered inconclusive. In PSP, AD and FTD groups, the percentages of positive CSF samples were lower (6.2%, 11.4%, and 28.6%, respectively), except for AD along with published rates of copathology [11, 18, 25]. With regard to the 12.5% positivity rate in our AD cohort, the prevalence of positivity aligns with previously published SAA results in these patients [41] and is lower than expected from post-mortem studies [11]. However, it has been shown that LBP occurs later than AD pathology and often as amygdala- or olfactory-predominant for which αSyn SAAs display lower sensitivity [28]. Clinical details for the positive PSP, AD, and FTD cases are provided in Supplementary Table 1. These cases include individuals with varying clinical characteristics, such as advanced disease stages in PSP cases, older age or cognitive decline in AD cases, and pathogenic mutations (e.g., TBK1, MAPT) in FTD cases. Due to the small number of positive cases, these observations remain descriptive and require further investigation in larger cohorts.

**Table 2:** CSF SAA test results of the study cohort at baseline

Clinical diagnosis	Samples tested positive [*n*]	Samples tested not positive [*n*]		Samples tested positive [%]	Samples tested not positive [%]
		Negative	Inconclusive		
PD/DLB	42 PD 3 DLB	0 PD 0 DLB	1 PD 0 DLB	97.8	2.2
MSA	0	30	1	0.0	100
CON	0	20	1	0.0	100
PSP	2	30	0	6.2	93.8
AD	4	29	2	11.4	88.6
FTD	2	5	0	28.6	71.4

*DLB* dementia with Lewy bodies, *AD* Alzheimer’s disease, *PD* Parkinson’s disease, *PSP* progressive supranuclear palsy, *MSA* multiple system atrophy, *CON* controls, *FTD* frontotemporal dementia, *n* number of individuals tested. CSF SAA test results were grouped into positive and non-positive categories (negative and inconclusive) and the respective percentages presented

The αSyn-SAA resulted in a 97.8% sensitivity against the Lewy-fold synucleinopathies PD and DLB

Lewy-fold specificity could be shown by a 100% specificity against MSA and controls

**Fig. 2:**
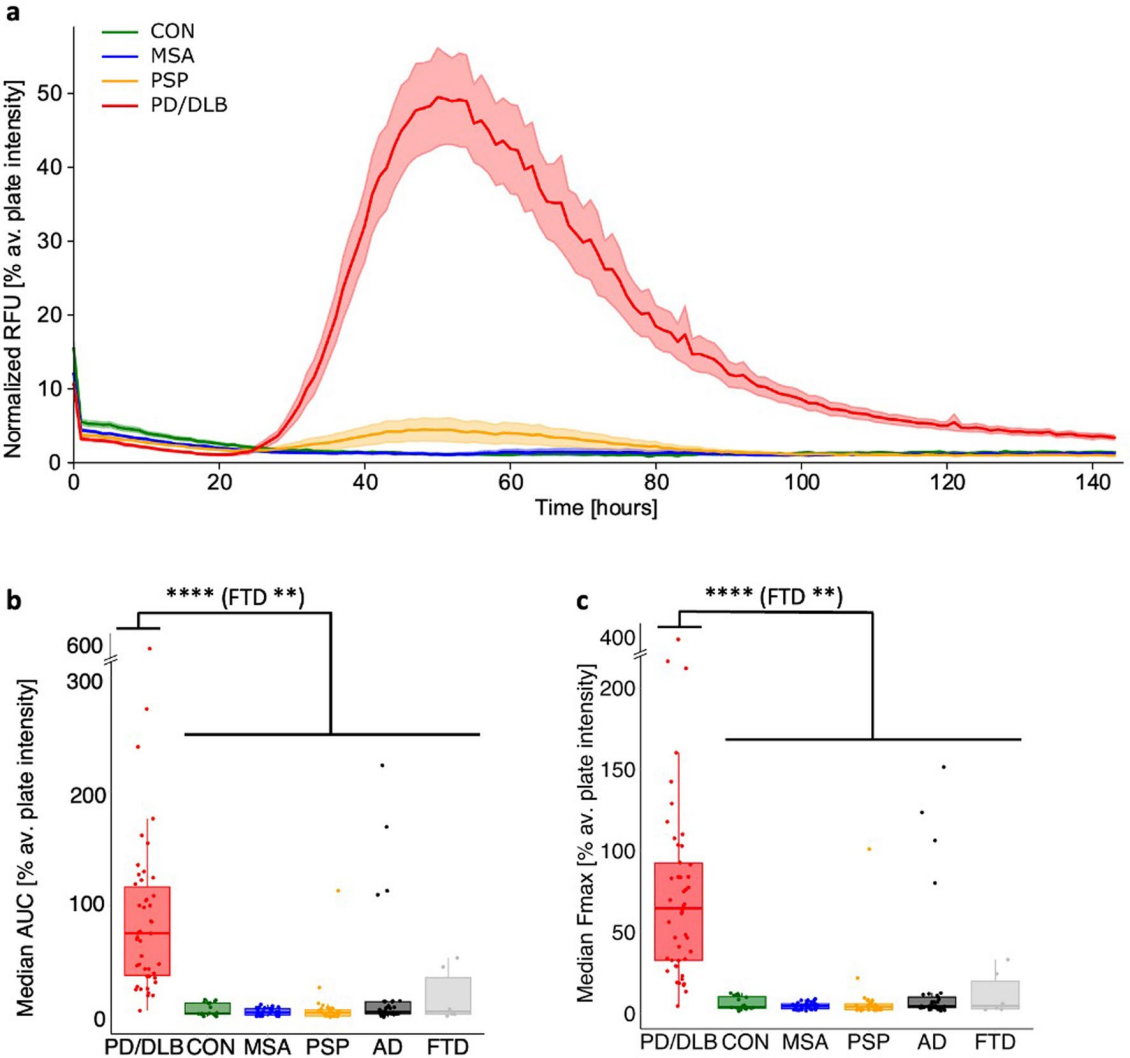
Comparison of CSF SAA results between PD/DLB and other neurodegenerative entities. *PD* Parkinson’s disease, *DLB* dementia with Lewy bodies, *CON* control, *MSA* multiple system atrophy, *PSP* progressive supranuclear palsy, *AD* Alzheimer’s disease, *FTD* frontotemporal dementia, *RFU* relative fluorescence units, *AUC* area under curve, *F*_*max*_ maximum fluorescence, *TTT* time to threshold, av.: average. **a** The curve characteristics of individual SAA reactions split for diagnoses are presented for PD/DLB (red, *n* = 46), MSA (blue, *n* = 31), PSP (yellow, *n* = 32), and CON (green, *n* = 20), highlighting the Lewy-fold specificity of the SAA for PD/DLB cases compared to no SAA reactions in MSA and CON cases that stay negative for 144 h. RFU values are normalized to the maximum intensity of fluorescence of the respective experimental plate. Each curve represents the average of the group ± standard error of the mean. **b** Comparison of the AUC of each group; each point depicts the median AUC for the replicates of the respective individual. **c** Comparison of the F_max_ of each group; each point depicts the median F_max_ for the replicates of the respective individual. Statistical analyses were conducted using Wilcoxon tests, resulting in a significance of *p* < 0.0001 (****) between PD/DLB against the other groups, except for FTD (** *p* ≤ 0.01)

The overlay of SAA reactions split for diagnoses (mean of the quadruplicate of each patient, normalized to maximum plate intensity to minimize inter-assay variations) of the cohort shows that Lewy-fold α-synucleinopathies display an increase in ThT signal, whereas MSA and CON CSF samples stay negative for the full 144 h (Fig. 2a). Several kinetic parameters can be derived from the time-resolved fluorescence data, including maximum fluorescence (F_max_), area under curve (AUC) and time to threshold (TTT). Both F_max_ and AUC metrics were significantly elevated in PD/DLB groups (Fig. 2b, c), with Wilcoxon tests confirming these differences (****p < 0.0001 for CON, MSA, PSP, AD and **p ≤ 0.001 for FTD).

To rule out the possibility that the αSyn SAA did not show αSyn seeding activity in MSA cases due to a lack of seeds in the CSF and to evaluate the Lewy-fold specificity in brain biosamples with neuropathologically confirmed αSyn pathology, the assay was utilized for the analysis of BH (frontobasal cortex and cerebellum) of α-synucleinopathy cases and controls. In contrast to MSA (*n* = 10) and non-α-synucleinopathy cases (CON *n* = 6, PSP *n* = 4), patients diagnosed with Lewy body diseases (LBD, *n* = 10) consistently showed early positive αSyn SAA signals, providing additional support for the assay’s specificity in identifying Lewy body pathologies (Supplementary Figures S2, S3). Demographic and neuropathological features of confirmed cases are presented in Supplementary Table 2.

Post-mortem brain tissue was available from 7 patients (3 MSA, 1 PSP, 1 AD—all without LBD copathology, 2 FTD cases with LBD copathology) of which CSF was tested ante-mortem as part of our series of 170 patients. Of note, the ante-mortem CSF SAA of all cases without LBD copathology was negative, whereas CSF in all cases with LBD copathology was positive, further corroborating the sensitivity and specificity of the assay (Supplementary Table 3).

### αSyn SAA quantification

**Fig. 3:**
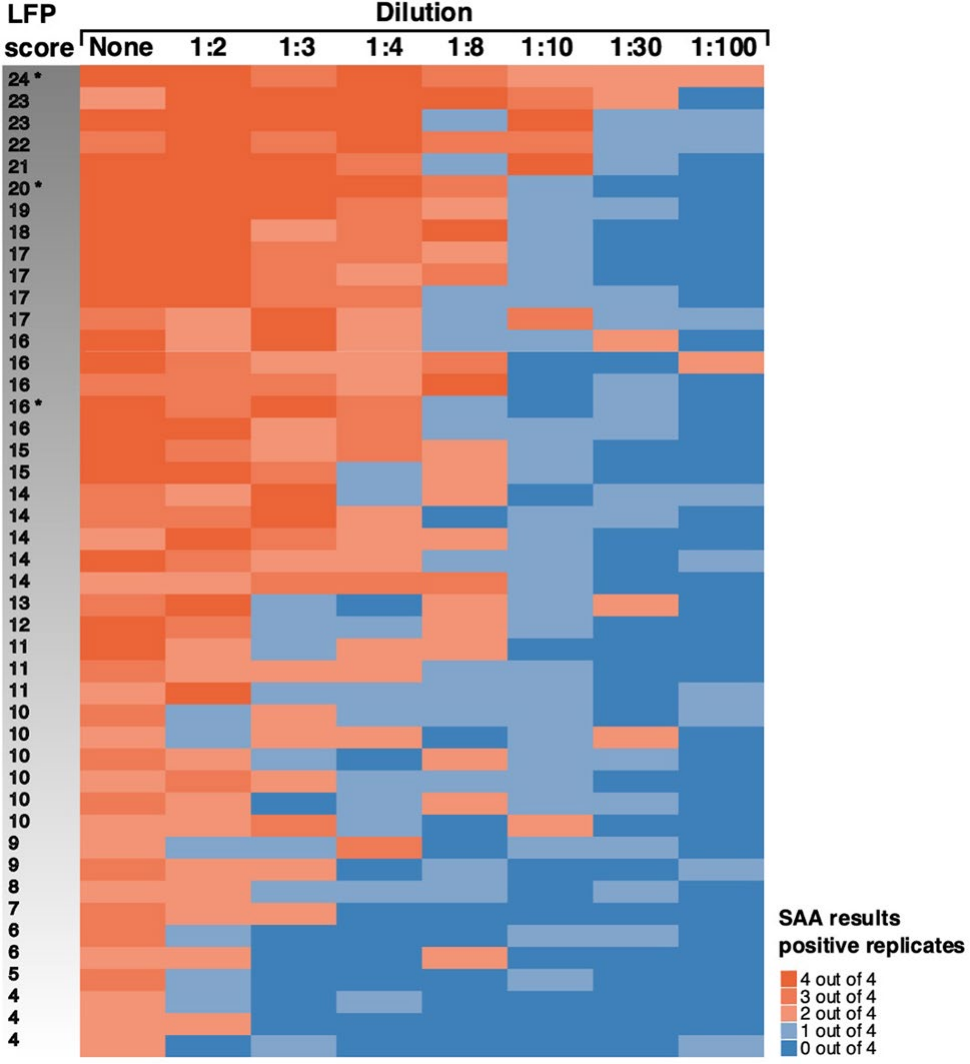
Dilution series for Lewy-fold pathology (LFP) score quantification. The 45 positively tested PD/DLB CSF samples (3 DLB, 42 PD) were serially diluted using negatively tested CSF and the number of positive signals was summed. We consider the total number of positive signals of this dilution series a surrogate marker for the Lewy-fold pathology and define it as the Lewy-fold pathology (LFP) score. The number of positive signals is shown in the left column. The samples are sorted by the number of positive signals. DLB patients are marked by asterisks (*). Number of positive replicates is presented by color code ranging from dark orange (4 out of 4) to dark blue (0 out of 4)

With the intention of developing a quantitative mode of analysis that might allow to draw conclusions about the initial seed load present in the CSF of the respective patient, we subjected αSyn SAA-positive samples to a dilution series with negatively tested CSF (Fig. 3). Two dilution series were employed: a twofold series with 1:2, 1:4, and 1:8 dilutions and a quasi-threefold series with 1:3, 1:10, 1:30, and 1:100 dilutions. This approach allowed stratification of the cohort based on the persistence of sample positivity in each dilution step: for example, some samples remained positive up to the 1:100 dilution (2 out of 45) and some became negative already at the 1:2 dilution (1 out of 45). This distribution showed that the two serial dilutions effectively covered the active range of seeding pathology.

**Fig. 4:**
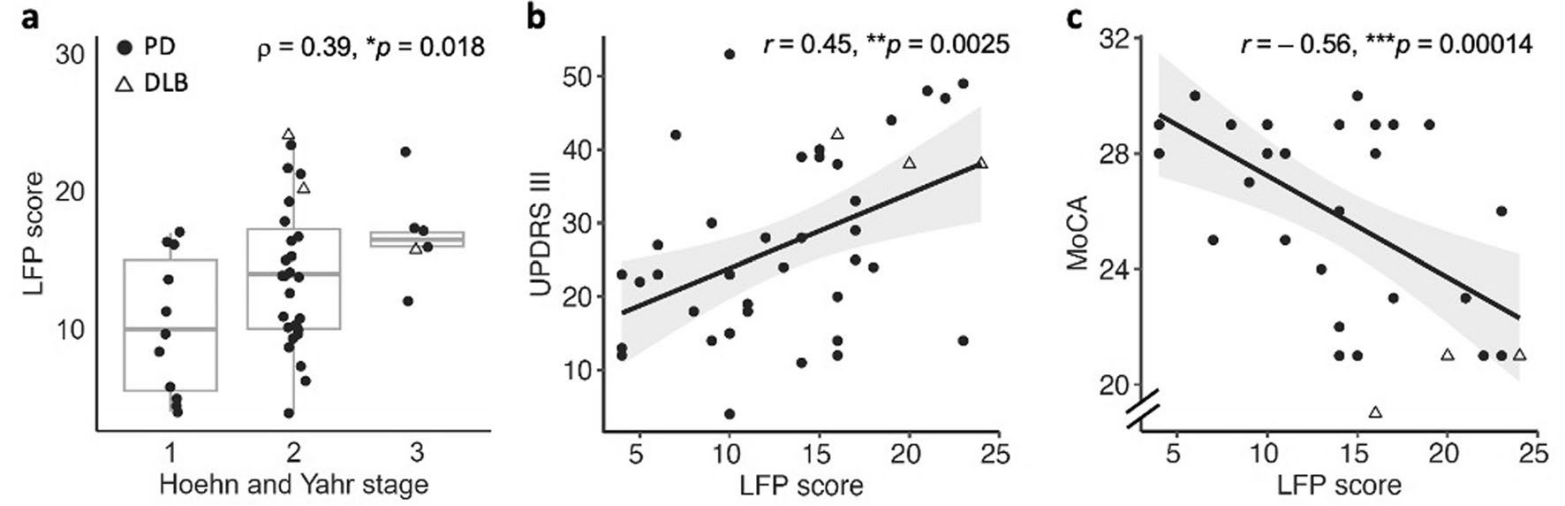
Lewy-fold pathology (LFP) score in association with disease severity scores. PD: Parkinson’s disease, DLB: dementia with Lewy bodies, MDS-UPDRS III: Movement Disorders Society Unified Parkinson’s Disease Rating Scale Part III, MoCA: Montreal Cognitive Assessment. In **a**–**c** bivariate associations between the clinical features Hoehn and Yahr stage (**a**), MDS-UPDRS III (**b**), and MoCA (**c**) and the LFP score are presented. The LFP score shows significant associations with the severity of disease (**a**, **b**) and cognition (**c**). Pearson (in **b**, **c**) and Spearman (**a**) correlation coefficients as well as *p* values corrected for age and sex are shown for each single association. In **b** and **c**), regression lines (black) and 95% confidence intervals (grey) are provided for continuous variables. For discrimination of diagnoses, PD and DLB samples are presented by dots and triangles, respectively

To gain a clearer understanding of the differences among the positive samples, we introduced the LFP score that was determined by summing the number of positive signals of the dilution series. The LFP score theoretically ranges from a minimum value of 2 (the minimal number of positive replicates of the neat CSF sample to be considered positive and qualify for dilution) to a maximum value of 32 (8 × 4 positive replicates), with the observed maximum being 24 (Fig. 3). Samples that stay positive at higher dilutions or have in general more positive replicates obtain higher LFP scores, most probably reflecting a higher seed load in the patient as underlying pathology. In contrast, a lower LFP score characterizes a patient with less pathological αSyn in the original sample. By stratifying each patient based on their individual LFP score, we investigated whether this score correlates with clinical features (Fig. 4). Specifically, the LFP score correlated with greater motor impairment as assessed by MDS-UPDRS III (Pearson’s *r* = 0.45, **p = 0.0025, corrected for age and sex) and H&Y stage (Spearman’s *ρ* = 0.39, *p = 0.018, corrected for age and sex) and with cognitive decline as measured by MoCA (Pearson’s *r* = – 0.56, ***p = 0.00014, corrected for age and sex). These correlations underscore the association between higher LFP scores and more advanced stages of PD as well as greater cognitive impairment, reflecting the spread of αSyn pathology to neocortical regions. The lacking associations between ATN biomarkers further support the concept that cognitive impairment in these patients is driven by αSyn pathology rather than amyloid or tau pathology (Supplementary Figure S4).

As an alternative method to derive quantitative information, we also examined kinetic parameters such as median TTT, AUC and F_max_ in the undiluted sample. However, these parameters did not show significant correlations with clinical features (Table 3, Supplementary Figure S5). Analysis of the median AUC revealed no significant correlations with clinical scores, including Hoehn and Yahr stage (*ρ* = 0.18, *p* = 0.58), MDS-UPDRS III (*r* = 0.18, *p* = 0.38), and MoCA (*r* = – 0.062, *p* = 0.74). Similarly, the median F_max_ showed no significant associations with these measures, with correlations of *ρ* = 0.22 (*p* = 0.46) for Hoehn and Yahr stage, *r* = 0.22 (*p* = 0.25) for MDS-UPDRS III, and *r* = – 0.14 (*p* = 0.45) for MoCA. In addition, we estimated the dilution producing 50% positive replicates to calculate the concentration of 50% seeding units (SD_50_) in the original CSF, providing a quantitative measure that should linearly correspond to the concentration of αSyn seeds by the Spearman–Kärber method [59]. Despite its quantitative potential, SD_50_ values showed weaker correlations with clinical features compared to LFP scores (Table 3, Supplementary Figure S6): H&Y (*ρ* = – 0.32, *p* = 0.056, corrected for age and sex), MDS-UPDRS III (*r* = – 0.34, *p = 0.029, corrected for age and sex) and MoCA (*r* = 0.53, ***p = 0.00071, corrected for age and sex). We, therefore, conclude that the LFP score is superior to SD_50_ in our experimental setting. This approach consumes a significant amount of resources, as only two patients can be fitted in one 96-well plate, which is suitable for comparing two time-points of the same patient. Omitting two dilutions allows to measure 4 samples on one 96-well plate. Thus, we tested if omitting the two highest dilutions (1:100, 1:30) changes the correlations between clinical parameters and the LFP score. The strength and significance of the correlations was only slightly reduced (Supplementary Table 4).

**Table 3:** Correlation of different SAA parameters with core clinical features

	LFP score		SD_50_		Median TTT		Median F_max_		Median AUC	
	Regression coefficient	*p* value	Regression coefficient	*p* value	Regression coefficient	*p* value	Regression coefficient	*p* value	Regression coefficient	*p* value
Hoehn and Yahr	0.39	0.0086	− 0.32	0.030	− 0.16	0.28	0.22	0.14	0.18	0.24
UPDRS III	0.45	0.0032	− 0.34	0.029	− 0.19	0.24	0.22	0.17	0.18	0.27
MoCA	− 0.56	0.00073	0.53	0.0015	0.21	0.24	− 0.14	0.44	− 0.062	0.73

*MDS-UPDRS III* movement disorders society unified parkinson’s disease rating scale part III, *MoCA* montreal cognitive assessment, *TTT* time to threshold, *AUC* area under the curve, *F*_*max*_ maximum fluorescence, *SD*_*50*_ 50% seeding dose, *LFP* lewy-fold pathology score

### Longitudinal analysis of LFP scores and disease progression

**Fig. 5:**
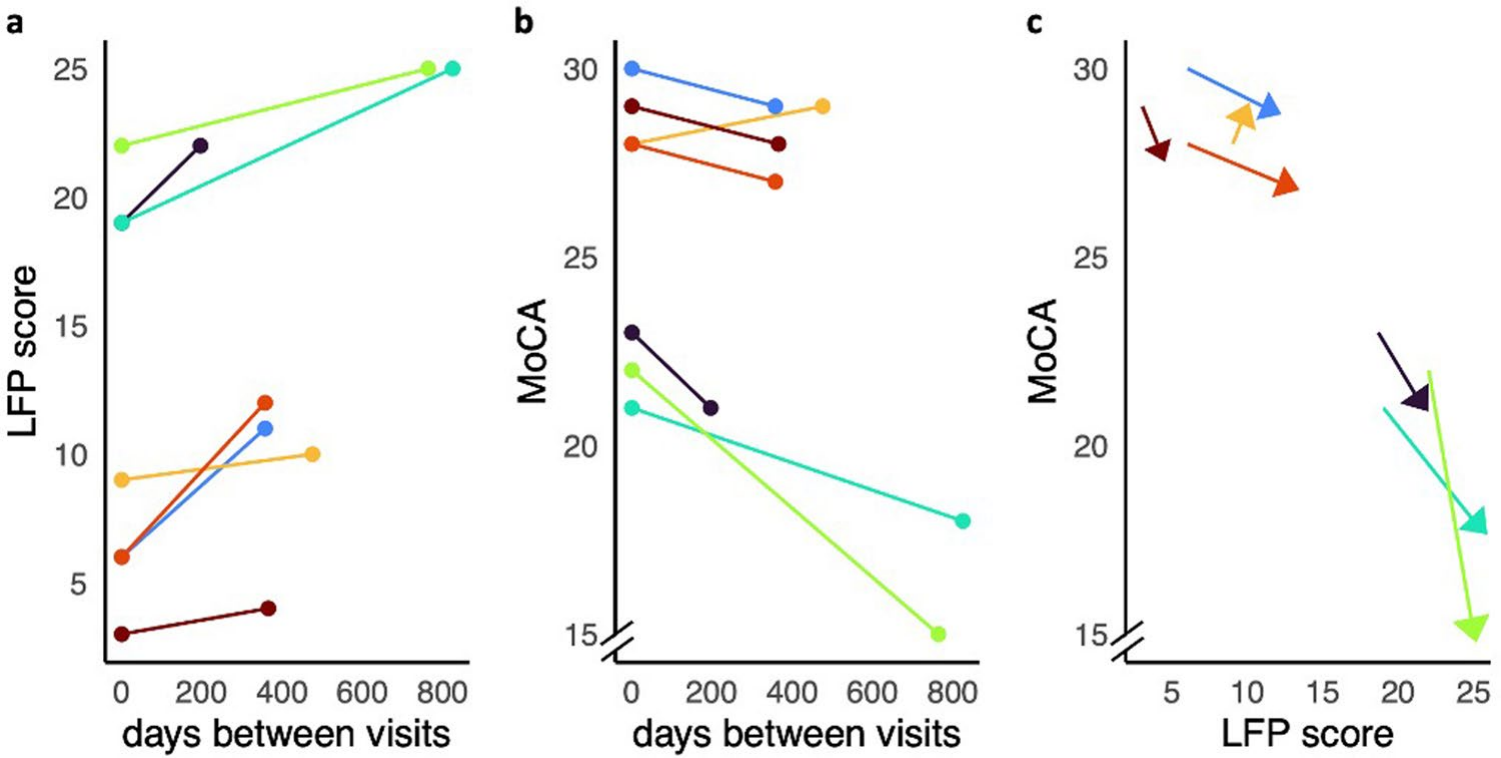
Longitudinal investigation of Lewy-fold (LFP) pathology score in 7 individuals with PD. PD: Parkinson’s disease, MoCA: Montreal Cognitive Assessment. LFP scores were determined of CSF of two serial lumbar punctures from 7 PD patients. LFP scores from the same patient are connected by a line. Each patient is shown in a specific color along **a**–**c** and associations with clinical measures of disease severity are presented. In **a**, the change of the LFP score over time is depicted, whereas **b** shows the corresponding change in MoCA. **c** aims to visualize the connection between an increase in LFP score as a measure for seed load and the decline in MoCA in follow-up samples compared to baseline evaluation LFP scores. Arrows connect baseline with follow-up values of each individual patient

To evaluate the potential of the LFP score as a marker for disease progression, we performed a longitudinal analysis on CSF samples collected from 7 PD patients over time (Fig. 5). Tracking LFP scores revealed progressive increases in all patients, which paralleled declining MoCA scores, except for one case where the MoCA score increased slightly from 28 to 29. Each patient consistently showed increasing LFP scores corresponding to worsening cognitive function. This finding underscores the potential of the LFP score to monitor disease progression over time at the individual level, not just at the group level. The consistently increasing LFP values over time further validate the quantitative potential of our approach. The relationship with motor disease severity scores is less evident, likely because these scores are influenced by dopaminergic therapy, which can mask underlying disease progression in motor symptoms (Supplementary Figure S7). Notably, all but one patient showed progressive increases in their levodopa dose over time.

## Discussion

In this study, we present a quantitative αSyn SAA with Lewy-fold specificity for CSF and brain homogenate samples. Blinded measurements demonstrated high sensitivity (97.8%) and specificity (100%) for Lewy-fold α-synucleinopathies, clearly distinguishing PD and DLB from MSA and detecting αSyn copathology in other neurodegenerative diseases such as AD and PSP. As highlighted by a recent consensus proposal for the biological definition of PD by Simuni et al. [50], there is a crucial need for quantitative biomarkers to measure disease progression and response to therapy. So far, assessments of αSyn SAAs provide only yes/no results. By counting the sum of all positive signals from a dilution series of positive samples and controls, we calculated a novel quantitative metric that we termed LFP score, which allowed us to stratify the patient cohort and to monitor disease progression at an individual level as revealed by longitudinal analyses. LFP scores showed significant correlations with clinical measures, including H&Y stage, MDS-UPDRS III, and MoCA.

The quality of the assay is further supported by the fact that CSF samples without pathological αSyn seeds remain negative throughout the measurement period (up to 6 days) as the intrinsic feature of αSyn to spontaneously aggregate is suppressed, preventing false-positive reactions in both neat samples and serial dilutions. In contrast, some laboratories terminate their fluorescence measurements earlier, e.g., after around 40 h, likely to avoid potential false-positive signals that may arise in controls. Interestingly, in our assay, the ThT signal exhibits an initial increase followed by a decline over time in positive samples. This phenomenon, also observed in other studies (e.g., Fig. 2 in Rossi et al. [41]) may reflect the natural decay of assay components.

The development of quantitative biomarkers for neurodegenerative diseases is critical for both diagnosis and therapeutic monitoring. While previous SAA approaches have primarily been used to provide binary outcomes (positive/negative) for diagnostic purposes, advancements in quantitation methodologies are beginning to expand their utility in tracking disease progression and evaluating treatment responses. Existing kinetic parameters, as maximum fluorescence (F_max_), time to threshold (TTT), time to 50% maximum (T_50_), second fastest TTT (TTT2), slope and area under the curve (AUC), have been explored in various studies [8–10, 13, 16, 33, 36, 48] but often fail to show consistent correlations with clinical severity, particularly in differentiating between stages of disease progression. A detailed overview of the available literature is presented in Supplementary Table 5. Of note, 2 publications compared different SAAs in the same study: Russo et al. [44] analyzed F_max_, AUC, TTT, T_50_ and endpoint dilution (SD_50_) across three different laboratories (AbbVie, Amprion and Caughey). AbbVie focused on the kinetic parameters F_max_, AUC and TTT. Significant correlations were found between these parameters and University of Pennsylvania Smell Identification Test (UPSIT) and MDS-UPDRS I scores. However, no consistent correlations with other clinical measures like MDS-UPDRS III or MoCA were observed. Amprion also analyzed F_max_, AUC and TTT, finding significant correlations with UPSIT scores. However, similar to the findings at Abbvie, no consistent correlations were observed with broader clinical measures. The Caughey laboratory took a more comprehensive approach, including SD_50_ to quantify relative amounts of seeding activity. They found positive correlations between SD_50_ and age (*r* =  + 0.36, *p* = 0.006), disease duration (*r* =  + 0.31, *p* = 0.02) and NfL levels (*r* =  + 0.51, *p* = 0.05). Despite these findings, no consistent correlations were observed between SD_50_ and clinical measures such as MDS-UPDRS III or MoCA. Kang et al. [27] focused on T_50_ across two different laboratories (Soto, Green). The Soto laboratory evaluated T_50_ values in a cohort of 100 PD patients. The study found no significant correlations between T_50_ values and disease characteristics such as H&Y stage (*R*^2^ = 0.0099, *p* = 0.3235), MDS-UPDRS III (*R*^2^ = 0.0013, *p* = 0.7202) and MDS-UPDRS total scores (*R*^2^ = 0.0004, *p* = 0.8458). The Green laboratory also evaluated T_50_ values in a cohort of 101 PD patients. Similar to the findings from the Soto laboratory, no significant correlations were found between T_50_ values and clinical measures including H&Y stage (*R*^2^ = 0.0093, *p* = 0.3365), MDS-UPDRS III (*R*^2^ = 0.0039, *p* = 0.5338) and MDS-UPDRS total scores (*R*^2^ = 0.0100, *p* = 0.3204). Recent advancements in SAA quantitation, such as those by Srivastava et al. [52], have demonstrated that employing endpoint dilution methods, combined with adjustments to dilution factors, replicate numbers, and analytical frameworks, can improve precision, allowing detection of smaller differences in αSyn seed concentrations. While these methodological improvements highlight the evolving nature of quantitative SAAs, the study does not report correlations with clinical measures. Our study introduces the LFP score as a robust, quantitative marker that correlates with established clinical scales such as MDS-UPDRS III and MoCA. Importantly, the LFP score can track disease progression over time, offering a more dynamic tool for monitoring αSyn pathology in PD and DLB. This feature makes it particularly valuable for clinical trials targeting αSyn aggregation, where precise monitoring of disease progression is essential for evaluating treatment efficacy.

There are several factors that may contribute to the inconsistency regarding clinical correlates of aSyn SAA parameters. One of the most important is the inter-individual variability of the CSF matrix that can speed up or slow down the reaction, shifting kinetic parameters such as the TTT and introducing noise, making TTT a variable measure. Several factors within the CSF matrix can accelerate αSyn aggregation. For instance, acidic pH enhances aggregation by exposing hydrophobic domains, while metal ions (e.g., Fe^2^⁺, Cu^2^⁺) neutralize charge repulsion and stabilize aggregation-prone structures [12]. Polyamines such as spermine, proteoglycans, nucleic acids (e.g., double-stranded DNA), poly-ADP ribose and fatty acids also promote aggregation by increasing local αSyn concentrations or facilitating membrane interactions. CSF matrix varies widely regarding lipoproteins and albumin. Albumin, the most abundant protein in blood and CSF, may inhibit the aggregation of various amyloidogenic proteins, including αSyn [1, 17, 26]. In addition, high-density lipoproteins in CSF can inhibit αSyn aggregation, indicating that the overall composition of CSF significantly impacts αSyn SAA kinetics [4]. This study by Bellomo et al. highlighted the need to consider intrinsic CSF components in interpreting kinetic parameters such as TTT, AUC, and F_max_ due to donor-dependent inhibitory effects on αSyn aggregation. The TTT for example depends heavily on seed concentration and reaction efficiency, which works well with fibrils in a consistent matrix [43, 49]. Furthermore, the calculation of TTT is not straightforward (as well as of F_max_ and AUC), especially when not all replicates in a quadruplicate are positive. The mean is sensitive to outliers, making the median a better measure; however, if only two or three replicates in a sample are positive and the others are negative, the TTT is infinite and a median or mean cannot be calculated reliably. Despite the possibility for LFP scores to produce imperfect data points, they provide comprehensive coverage of the positive signals across the dilution series. Using 8 dilutions (including neat) with 4 replicates each captures a dynamic range of signals, effectively covering 32 points. Employing two dilution series (e.g., 1:2, 1:4, 1:8 and 1:3, 1:10, 1:30, and 1:100) ensures better resolution in the relevant ranges and reliable results.

A further advantage of the serial dilution approach lies in the standardization of the CSF matrix as the original biosample is increasingly diluted in standardized control CSF. Noteworthy, the occurrence of positive SAA signals is more variable than expected, occasionally resulting in the reappearance of positive replicates at higher dilutions. This may be due to several factors, such as heterogeneity of seeds in patients. SAAs are inherently non-linear, and slight variations in initial conditions—such as seed concentration, seed conformation and additional proteins or small molecules—can significantly impact the aggregation kinetics or even prevent successful aggregation.

Our findings suggest that the LFP score likely detects the spread of pathology and/or the amount of αSyn aggregates in the CNS rather than the aggressiveness of the disease. By “aggressiveness”, we refer to the rate of clinical progression, defined by how rapidly symptoms such as motor impairment (e.g., MDS-UPDRS III, Hoehn and Yahr stages) or cognitive dysfunction (e.g., MoCA) worsen over time. MDS-UPDRS III, H&Y (motor dysfunction) and MoCA (cognitive dysfunction) scores correlate significantly with the LFP score (Fig. 4). These clinical measures represent how widely the pathology is distributed in the brain. However, our analysis showed (Supplementary Figure S8) that the LFP score was not significantly associated with disease duration (*r* = 0.17, *p* = 0.274), changes in MDS-UPDRS III over 12 months (*r* = 0.26, *p* = 0.095), changes in levodopa daily dose over 12 months (*r* = 0.075, *p* = 0.620), or changes in MoCA scores over 12 months (*r* =  0.039, *p* = 0.831). These findings suggest that the LFP score reflects a pathological characteristic rather than clinical progression or aggressiveness. We are the first to present an SAA that tracks the progression of αSyn pathology at an individual level in CSF, as previous studies could not consistently show changes in SD_50_ values or other kinetic parameters between paired baseline and follow-up samples [44]. The LFP score increased over time in all seven individuals studied (Fig. 5), likely indicating cumulative αSyn pathology progression in the CNS. This observation is consistent with Braak’s staging framework, which correlates disease progression with the spread of αSyn pathology [7]. However, the progression of the disease does not necessarily become more aggressive over time. This notion is consistent with a recently published study [5] which found that the sensitivity of SAAs correlated with the extent of Lewy body pathology, showing higher sensitivity in advanced stages of LBD. The study also highlighted that the number of positive SAA replicates correlated with the αSyn pathology burden. While no validated measures of disease-associated αSyn species in CSF are currently available for direct comparison, indirect evidence from Braak’s framework and recent studies supports the LFP score as a measure of pathology burden rather than clinical aggressiveness.

Our data, in conjunction with findings from recent studies, strongly support the hypothesis that PD, DLB, and MSA are linked to distinct strains of pathological αSyn aggregates. Recent cryo-electron microscopy studies [46, 60] provided detailed structural insights into αSyn fibrils from different α-synucleinopathies. It was shown that αSyn fibrils from MSA patients have unique structural characteristics distinct from those in PD and DLB. The αSyn filaments from PD and DLB share an identical Lewy-fold, characterized by a single protofilament structure. This Lewy-fold is markedly different from the αSyn filaments found in MSA. In MSA, there are two distinct filament types, each made up of two different protofilaments. Even though it is still unclear what pathological protein species is present in the CSF with low-*n* oligomers being a plausible candidate, our results as well as other publications [47] suggest that the seeds keep the molecular characteristic of their respective underlying α-synucleinopathy. These structural differences likely influence the seeding properties of the aggregates. The binding and nucleation of recombinant αSyn monomers in amplification assays are highly dependent on the molecular characteristics of the seeds. While our assay is optimized for detecting Lewy-related αSyn aggregates, specific conditions (e.g., pH, salt concentrations, and detergent composition) required for amplifying MSA strains may not be present in our setup. For example, studies suggest that MSA-specific strains may require lower pH, higher salt concentrations, or alternative shaking parameters to achieve efficient amplification [47].

Our study has several limitations. First, the results predominantly reflect a Caucasian population in Germany, which may restrict the applicability of our findings to other ethnic groups. Patients were recruited from a single tertiary center only (LMU Munich). To minimize the risk of clinical misdiagnosis, all patients were evaluated at a specialized outpatient clinic for movement disorders, and diagnoses were reconfirmed at each follow-up visit. We have not yet assessed the application of our assay in prodromal stages, such as idiopathic REM sleep behavior disorder (iRBD). Existing data suggest that SAAs can detect α-synuclein pathology in prodromal stages [24], making this an important direction for future research. While CSF sampling remains invasive, less invasive approaches, such as peripheral tissue sampling, may facilitate broader applicability in early detection and stratification frameworks.

The novel LFP score exhibits strong correlations with clinical severity measures, establishing it as a promising progression marker for Lewy-fold α-synucleinopathies such as PD and DLB and differentiating these conditions from MSA in blinded measurements. The findings underscore the significance of dilution series and the resulting LFP score in accurately capturing the extent of αSyn pathology across the central nervous system. Notably, our study introduces a novel aspect as our SAA is not only quantitative but also capable of capturing disease progression longitudinally at an individual level which sets it apart from previous approaches. This quantitative capability positions our SAA in combination with the LFP score as a potential pharmacodynamic/response tool for evaluating new disease-modifying therapies targeting αSyn, thereby offering significant opportunities in clinical trials and therapeutic monitoring. Future research should aim to validate these results in larger and longitudinal clinical cohorts to enhance their generalizability and clinical applicability.

## Supplementary Information

Below is the link to the electronic supplementary material.

Supplementary file1 (PDF 2765 KB)

## Data Availability

The raw data used in preparation of the figures and tables will be shared in anonymized format upon reasonable request in agreement with EU legislation on the general data protection regulation and be regulated in a material transfer agreement.

## Abbreviations

AD: Alzheimer’s disease
AUC: Area under the curve
ATN: Amyloid tau neurodegeneration
BH: Brain homogenate
CON: Controls
CSF: Cerebrospinal fluid
CNS: Central nervous system
DLB: Dementia with Lewy bodies
F_max_: Maximal fluorescence intensity
FTD: Frontotemporal dementia
LFP: Lewy-fold pathology
LBD: Lewy body disease
LP: Lumbar puncture
MDS-UPDRS: Movement disorder society unified parkinson’s disease rating scale
MoCA: Montreal cognitive assessment
MSA: Multiple system atrophy
PBS: Phosphate-buffered saline
PD: Parkinson’s disease
PMCA: Protein misfolding cyclic amplification
PSP: Progressive supranuclear palsy
PrP^Sc^: Prion protein scrapie
qRT-QuIC: Quantitative real-time quaking-induced conversion
RFU: Relative fluorescence units
αSyn: Alpha-synuclein
SAA: Seed amplification assay
SD_50_: 50% Seeding dose
TTT: Time to threshold
T_50_: Time to 50% maximal fluorescence
UMSARS: Unified multiple system atrophy rating scale
UPSIT: University of pennsylvania smell identification test
