# Supplementary material for: A quantitative Lewy-fold-specific alpha-synuclein seed amplification assay as a progression marker for Parkinson’s disease
Source: Acta Neuropathol. 2025 Feb 20;149(1):20. doi: 10.1007/s00401-025-02853-y (PMC11842418; doi:10.1007/s00401-025-02853-y)
Supplement: Supplementary file 1 — Supplementary file1 (PDF 2765 KB) [file 401_2025_2853_MOESM1_ESM.pdf]

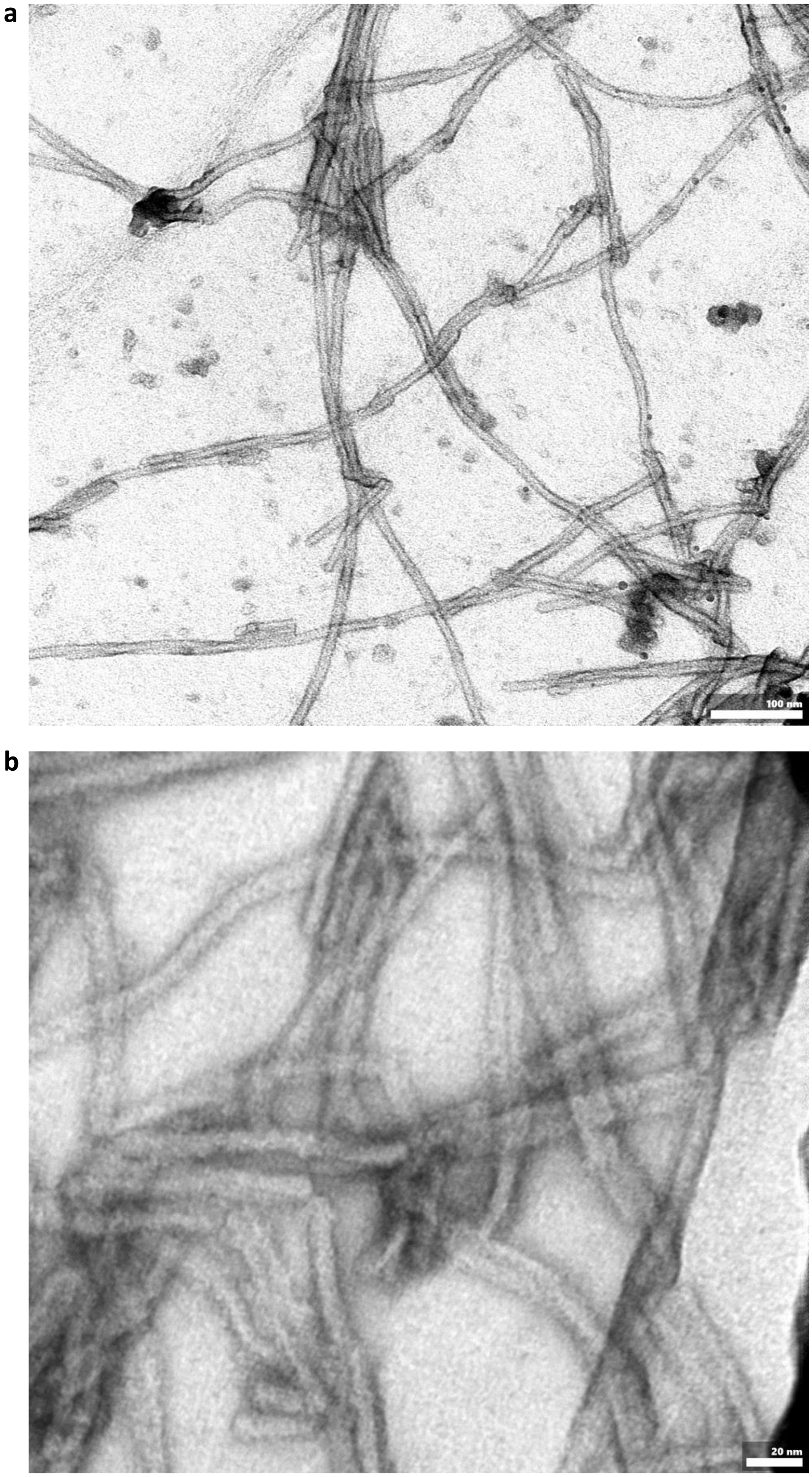

Supplementary Figure S1. Electron micrographs of in vitro produced αSyn fibrils

Electron micrographs of in vitro produced αSyn fibrils are shown. (**a**) Scale bar: 100 nm; (**b**) Scale bar: 20 nm. For transmission electron microscopy (TEM), carbon-coated copper grids (Science Services) were glow-discharged for 30 seconds using a Harrick plasma cleaner (PDC-32G-2) to facilitate adsorption. A 1.5 µl sample was deposited onto the grid for 2 minutes using anti-capillary inverse tweezers (Dumont), blotted briefly with filter paper (Whatman), and negatively stained with 1% uranyl acetate in water for 30 seconds. After blotting, grids were air-dried for at least 30 minutes. Micrographs were acquired using a JEM 1400plus TEM (JEOL) equipped with a XF416 camera (TVIPS) and EM-Menu software (TVIPS).

#	Diagnosis	Subtype	Age at LP	disease duration	sex	Pathogenic genetic mutation	MDS-UPDRS III at LP	Hoehn and Yahr at LP	MOCA at LP
**1**	AD	amnestic	57	3	w	NA	NA	NA	9
**2**	AD	amnestic	76	5	w	NA	NA	NA	21
**3**	AD	amnestic	76	3	m	NA	NA	NA	14
**4**	AD	CBS	77	1	w	NA	NA	NA	8
**5**	FTD	bvFTD	63	5	w	MAPT	NA	NA	17
**6**	FTD	bvFTD	74	3	w	TBK1	NA	NA	21
**7**	PSP	P	79	3	m	NA	35	3	NA
**8**	PSP	PSP-RS	82	3	m	NA	55	4	NA

Supplementary Table 1. Demographic and clinical characteristics of CSF αSyn SAA-positive AD, FTD and PSP cases

PSP: Progressive supranuclear palsy, FTD: Frontotemporal dementia, AD: Alzheimer’s disease, CBS: Corticobasal syndrome, P: PSP-parkinsonism, PSP-RS: Richardson's syndrome, bvFTD: Behavioral variant frontotemporal dementia, NA: Not applicable, m: male, f: female, UPDRS III: Unified Parkinson’s Disease Rating Scale Part III, H&Y: Hoehn and Yahr stage, MoCA: Montreal Cognitive Assessment.

#	Sex	Age	Clinical Diagnosis	Neuropathological Diagnosis	Post mortem interval [hours]	LBD Braak	AD Braak	Amyloid Thal
**1**	f	79	PD	LBD	42	6	2	0
**2**	f	80	PD	LBD	33	6	2	0
**3**	m	75	PD	LBD	NA	6	1	1
**4**	f	74	PD	LBD	22	6	1	1
**5**	m	82	PD	LBD	23	6	2	5
**6**	f	79	PDD	LBD	26	6	4	3
**7**	m	77	PD/DLB	LBD	34	6	4	4
**8**	f	84	PDD	LBD	59	6	3	5
**9**	m	76	DLB	LBD	NA	6	4	4
**10**	m	68	DLB	LBD	37	6	3	4
**11**	m	68	MSA	MSA	61	0	1	0
**12**	f	52	MSA-P	MSA	81	0	1	1
**13**	f	64	MSA-P	MSA	52	0	1	2
**14**	f	62	Parkinsonian syndrome	MSA	72	0	1	0
**15**	m	64	MSA-P	MSA	22	0	1	0
**16**	f	54	MSA-P	MSA	50	0	1	2
**17**	f	75	MSA-P	MSA	73	0	1	1
**18**	m	69	MSA-P	MSA	10-34	0	1	1
**19**	m	70	MSA-P	MSA	24	0	3	0
**20**	m	68	MSA-P	MSA	41	0	2	0
**21**	f	64	NA	Control	33	0	1	0
**22**	m	58	NA	Control	22	0	1	0
**23**	f	64	NA	Control	39	0	1	0
**24**	m	67	NA	Control	25-31.5	0	1	2
**25**	m	51	NA	Control	24	0	0	3
**26**	f	66	Control	Control	27-39.8	0	1	3
**27**	f	79	PSP	PSP	16	0	2	1
**28**	m	61	PSP	PSP	72	0	0	0
**29**	m	67	PSP	PSP	38	0	1	1
**30**	m	65	PSP	PSP	33	0	0	0

Supplementary Table 2. Demographic and neuropathological features of neuropathologically confirmed cases

LBD: Lewy body disease, DLB: Dementia with Lewy bodies, AD: Alzheimer's disease, PD: Parkinson's disease, PDD: Parkinson's Disease Dementia, PSP: progressive supranuclear palsy, MSA: multiple system atrophy, NA: Not applicable, m: male, f: female

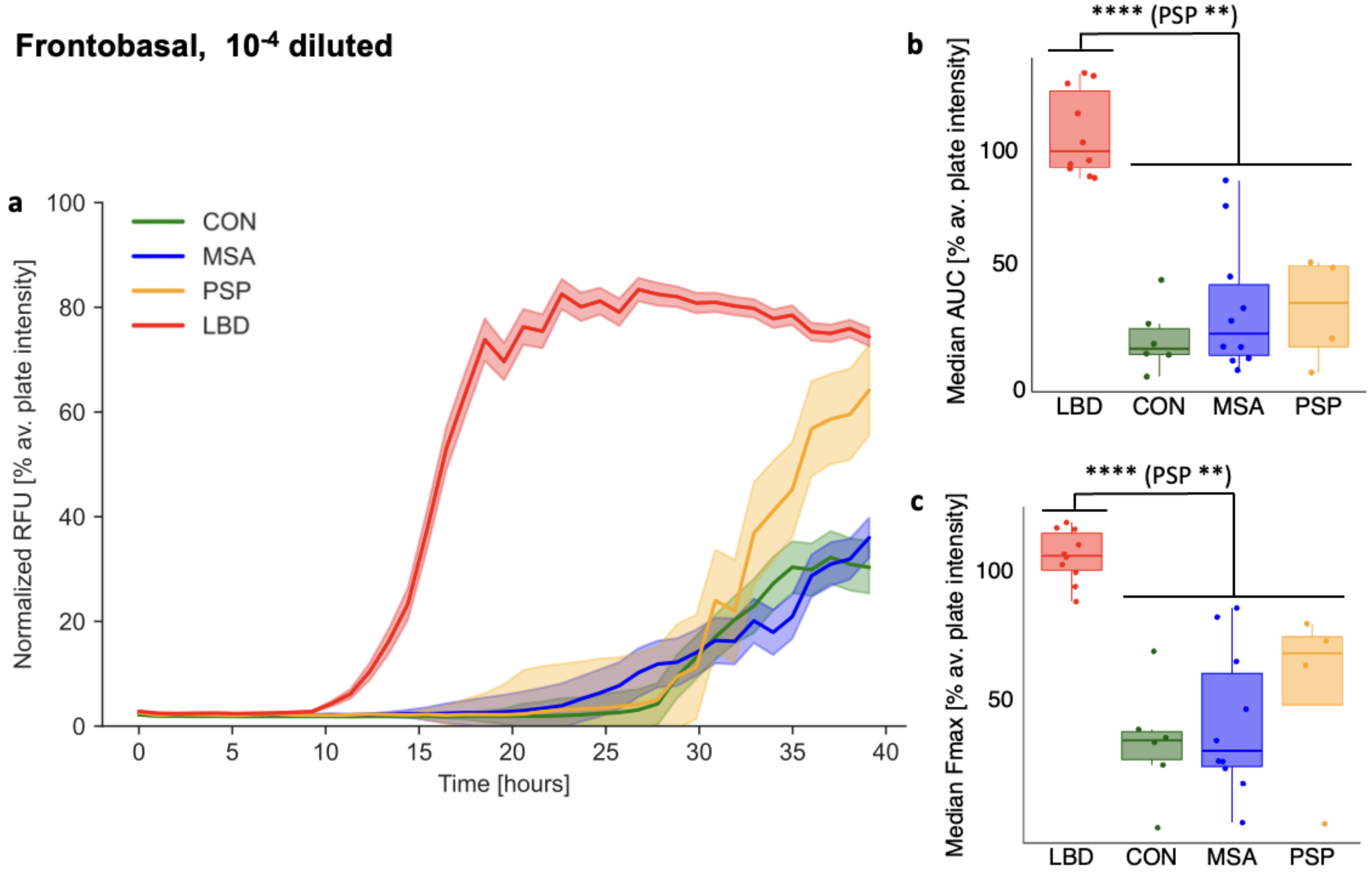
Frontobasal, 10^{-4} diluted
a
CON
MSA
PSP
LBD
Normalized RFU [% av. plate intensity]
Time [hours]
b
**** (PSP **)
Median AUC [% av. plate intensity]
LBD
CON
MSA
PSP
c
**** (PSP **)
Median Fmax [% av. plate intensity]
LBD
CON
MSA
PSP

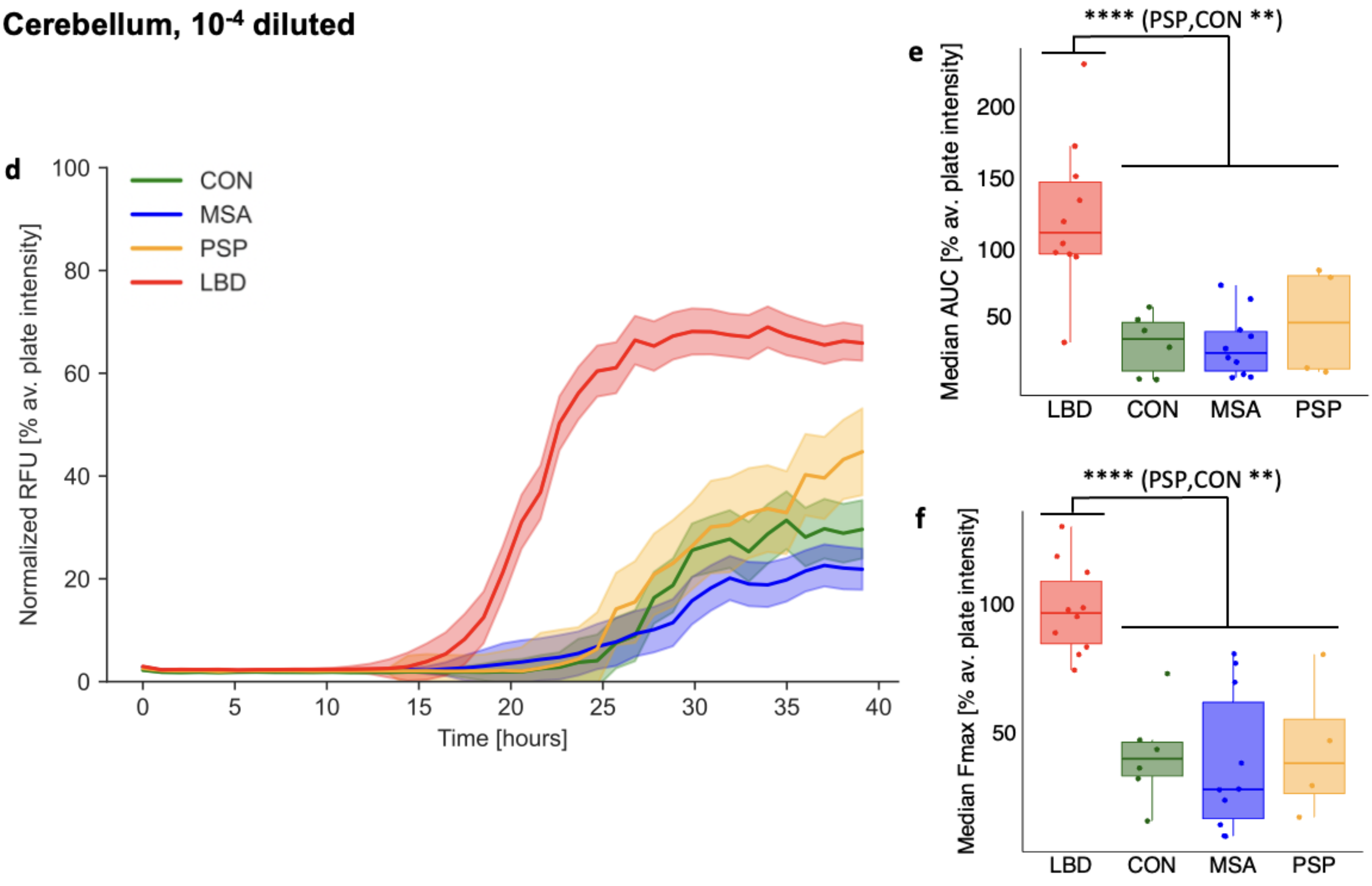
Cerebellum, 10^{-4} diluted
d
CON
MSA
PSP
LBD
Normalized RFU [% av. plate intensity]
Time [hours]
e
**** (PSP,CON **)
Median AUC [% av. plate intensity]
LBD
CON
MSA
PSP
f
**** (PSP,CON **)
Median Fmax [% av. plate intensity]
LBD
CON
MSA
PSP

Supplementary Figure S2. Detection of αSyn seeding activity in brain homogenates from cases with LBD but not MSA and non-synucleinopathy cases

LBD: Lewy-body disease, PSP: progressive supranuclear palsy, MSA: multiple system atrophy, RFU: Relative fluorescence units, av.: average.

In **a)** and **d)** each curve represents the average of the group. Error bars indicate the standard error of the mean. RFU values are normalized to percentage of the maximum intensity of fluorescence of the respective experimental plate. Comparison among brain homogenates (PBS soluble fraction, 10% w/v) from LBD (red line, *n* = 10), MSA (blue line, *n* = 10), PSP (yellow line, *n* = 4), and CON (green line, *n* = 6) from frontobasal cortex and white matter (**a**) and cerebellum (**d**). 2 µl of a 10^{-4} dilution of the respective brain homogenates were used alongside 98 µl reaction buffer. **b)** Comparison of the AUC of each group from frontobasal cortex; each point depicts the median AUC for the replicates of the respective individual. **c)** Comparison of the F_{max} of each group from frontobasal cortex; each point depicts the median F_{max} for the replicates of the respective individual. **e)** Comparison of the AUC of each group from cerebellum; each point depicts the median AUC for the replicates of the respective individual. **f)** Comparison of the F_{max} of each group from cerebellum; each point depicts the median F_{max} for the replicates of the respective individual. Statistical analyses were conducted using Wilcoxon tests, resulting in a significance of $p < 0.0001$ (****) between LBD against the other groups, except for PSP from frontobasal cortex as well as PSP and CON from cerebellum (all ** $p \leq 0.01$). The late-phase increase in fluorescence observed in MSA, PSP, and control samples (CON) likely reflects de novo aggregation of αSyn influenced by components in the brain homogenate, rather than spontaneous aggregation of monomeric αSyn in isolation.

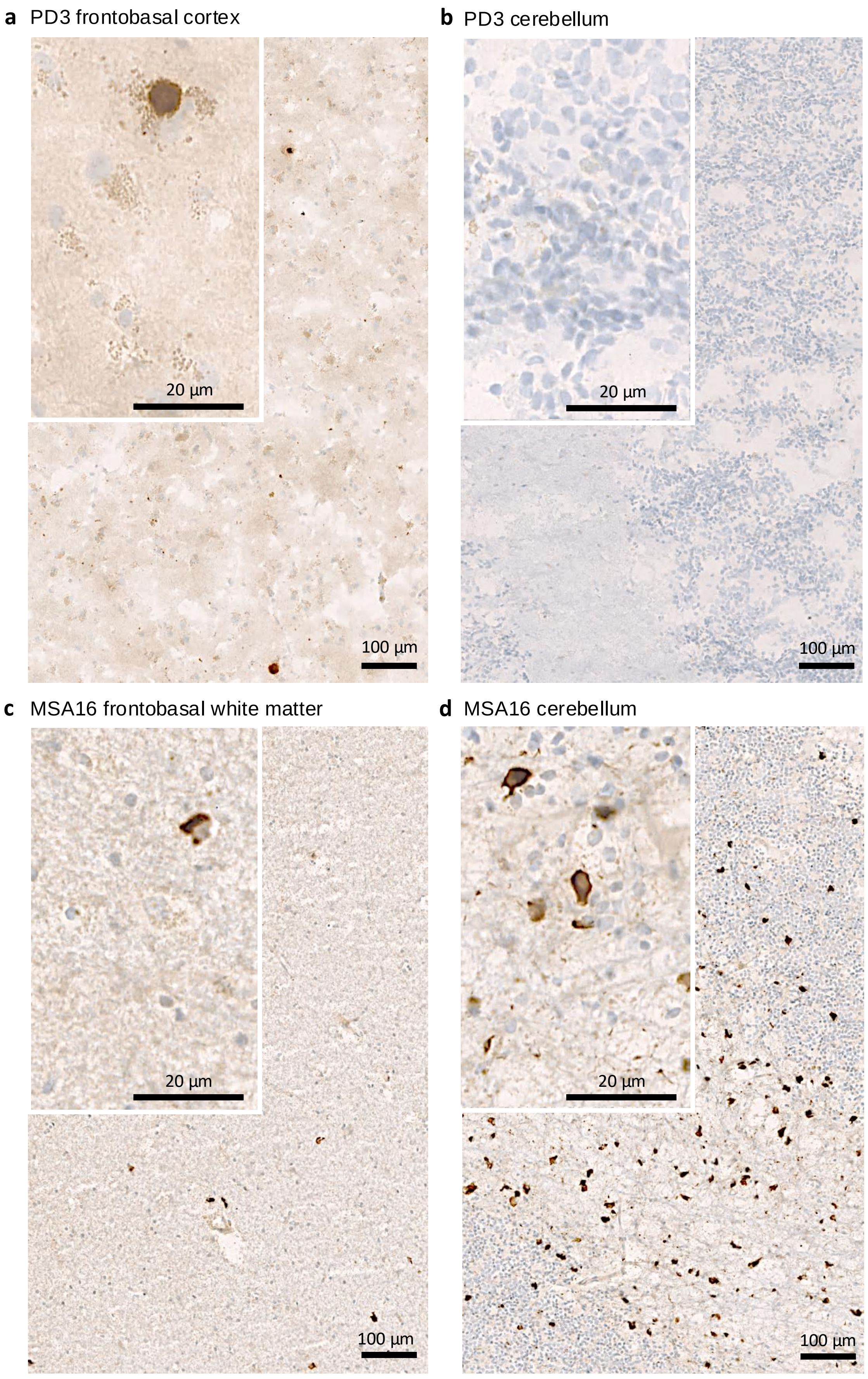

a PD3 frontobasal cortex
20 µm
100 µm
b PD3 cerebellum
20 µm
100 µm
c MSA16 frontobasal white matter
20 µm
100 µm
d MSA16 cerebellum
20 µm
100 µm

Supplementary Figure S3. αSyn pathology in representative patients with LBD and MSA

αSyn was stained by the Clone 42 antibody in fresh frozen brain tissue. Adjacent sections of the same fresh frozen tissues were used for producing PBS-soluble fractions for the SAA (see supplementary figure S1). **a**) Low number of Lewy-bodies in the frontobasal cortex of a case with LB pathology (case #3). **b**) No visible Lewy-bodies in the cerebellum of a case with LB pathology (case #3). **c**) Some glial cytoplasmic inclusions (GCIs) in oligodendroglia in the frontobasal white matter of a patient with MSA (case #16). **d**) Numerous glial cytoplasmic inclusions (GCIs) in oligodendroglia in the cerebellum of a patient with MSA (case #16).

#	Sex	Age at LP	Age at Death	Clinical Diagnosis	Primary NP diagnosis	Secondary NP diagnosis	SAA result (in CSF)
**1**	m	62	63	MSA-P	MSA	None	Negative (0/4)
**2**	m	55	58	MSA-C	MSA	None	Negative (0/4)
**3**	m	53	56	MSA-C	MSA	AGD (Saito 1)	Negative (0/4)
**4**	f	63	64	Behavioral Variant FTD, MAPT pathological variant	FTLD (Pick's Disease, 3R-Tau)	LBD (Braak 3, McKeith Brainstem)	Positive (2/4)
**5**	f	74	75	Behavioral Variant FTD, TBK1 pathological variant	TDP-43 (Type A, Mackenzie et al. 2011)	LBD (Braak 5, McKeith Limbic), AGD (Saito 4), ARTAG, AD (Braak 2, Thal 0, CERAD 0, NIA: A0B1C0)	Positive (2/4)
**6**	f	75	77	PSP-RS	FTLD (PSP, 4R-Tau)	AD (Braak I, Thal 3, CERAD 0, NIA A2B1C0), ARTAG, AGD, no LBD pathology	Negative (0/4)
**7**	f	85	86	AD, amnestic subtype	AD (Braak VI, Thal 5, CERAD B, NIA A3B3C2)	CAA (Type I, Thal 3), TDP-43 (Josephs 5, Nelson 2), ARTAG, no LBD pathology	Negative (0/4)

Supplementary Table 3. Demographic and neuropathological features of definite cases with ante mortem CSF available

AD: Alzheimer's Disease, AGD: Argyrophilic Grain Disease, ARTAG: Aging-Related Tau Astrogliopathy, CAA: Cerebral Amyloid Angiopathy, CERAD: Consortium to Establish a Registry for Alzheimer's Disease, FTD: Frontotemporal dementia, FTLD: Frontotemporal lobar degeneration, LBD: Lewy body disease, LP: lumbar puncture, MSA-C: multiple system atrophy cerebellar type, MSA-P: multiple system atrophy parkinsonian type, NIA: National Institute on Aging, NP: Neuropathology, PD: Parkinson's disease, PSP: progressive supranuclear palsy, PSP-RS: progressive supranuclear palsy Richardson's syndrome, m: male, f: female

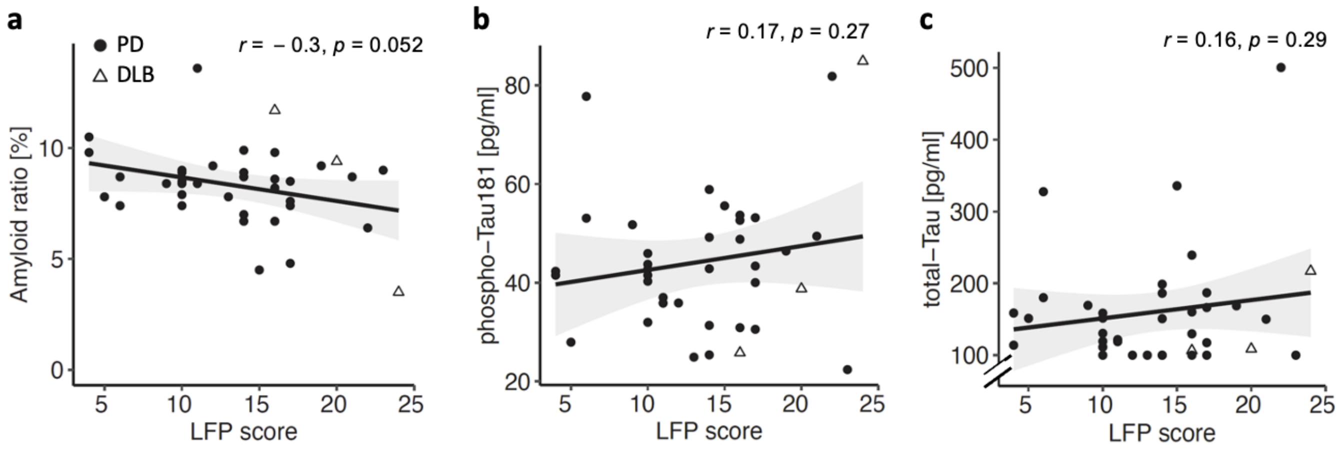

Supplementary Figure S4. Lewy-Fold Pathology (LFP) score in association with selected biomarker features

PD: Parkinson's disease, DLB: dementia with Lewy-bodies

In **a)** – **c)**, bivariate associations between selected biomarker features and the number of positive replicates in the dilution series, the LFP score, are presented. No significant correlations were found. Pearson's correlation coefficients as well as *p*-values corrected for age and sex are shown for each single association, regression lines (black) and 95% confidence intervals (grey) are provided for continuous variables. For discrimination of diagnoses, PD and DLB samples are presented by dots and triangles, respectively.

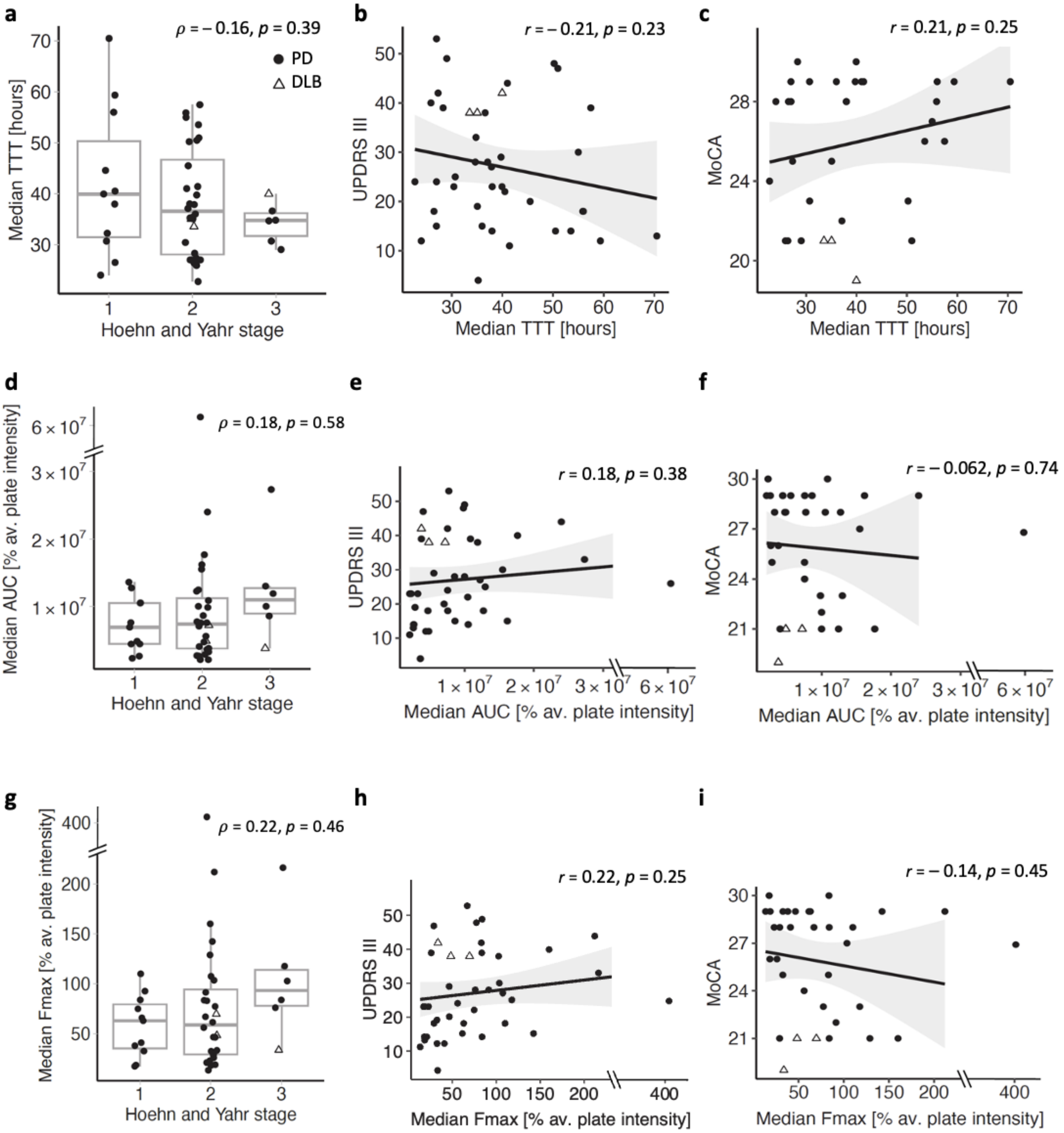

Supplementary Figure S5. Median time to threshold (TTT), area under the curve (AUC) and maximum fluorescence (F_{max}) in association with clinical and analytical features.

PD: Parkinson's disease, DLB: dementia with Lewy-bodies, MDS-UPDRS III: Movement Disorders Society Unified Parkinson's Disease Rating Scale Part III, MoCA: Montreal Cognitive Assessment, av.: average

In **a) – i)**, bivariate associations between clinical features, and the median TTT, AUC, and F_{max} are presented. Pearson (**b**, **e**, **f**, **h**, **i**) and Spearman (**a**, **d**, **g**) correlation coefficients as well as *p*-values corrected for age and sex are shown for each single association. Regression lines and 95% confidence intervals are provided for continuous variables (**b, c, e, f, h, i**).

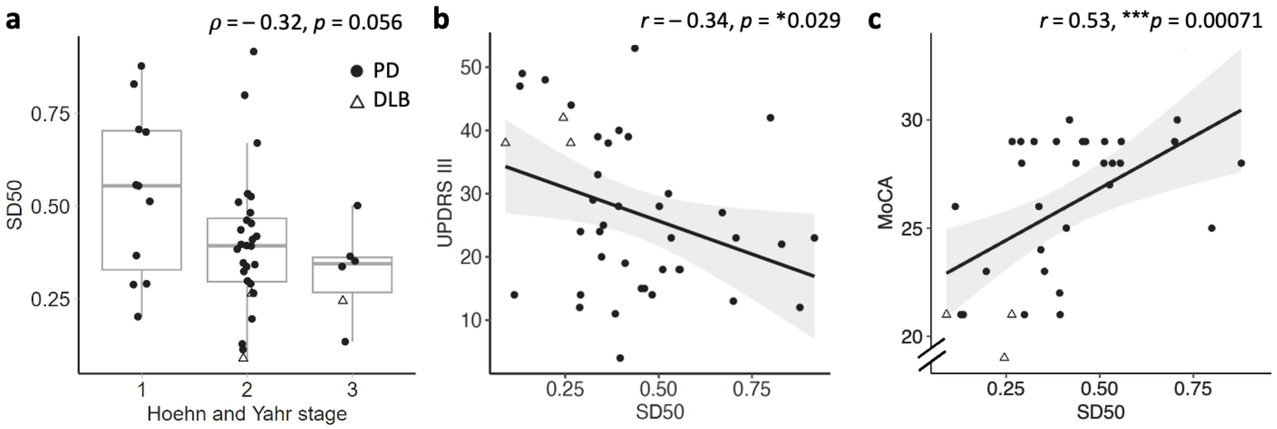

Supplementary Figure S6. 50% seeding dose (SD_{50}) in association with disease severity scores

PD: Parkinson's disease, DLB: dementia with Lewy-bodies, MDS-UPDRS III: Movement Disorders Society Unified Parkinson's Disease Rating Scale Part III, MoCA: Montreal Cognitive Assessment

The Spearman-Kärber method was used to calculate the sample dilution containing the number of seeds giving 50% positive replicate reactions, i.e., the 50% seeding dose or SD_{50}. In **a)** – **c)** bivariate associations between clinical features Hoehn and Yahr stage (**a**), MDS-UPDRS III (**b**), and MoCA (**c**) and the SD_{50} are presented. Pearson (in **b**, **c**) and Spearman (**a**) correlation coefficients as well as *p*-values corrected for age and sex are shown for each single association, in **b** and **c**) regression lines (black) and 95% confidence intervals (grey) are provided for continuous variables. For discrimination of diagnoses PD and DLB samples are presented by dots and triangles, respectively.

	LFP score (all dilutions)		LFP score without 1:100 and 1:30 dilution	
	Regression coefficient	p value	Regression coefficient	p value
**Hoehn and Yahr**	0.39	**0.018**	0.38	**0.056**
**MDS-UPDRS III**	0.45	**0.0025**	0.42	**0.029**
**MoCA**	-0.56	**0.00014**	-0.5	**0.00071**

Supplementary Table 4. Truncated Lewy-Fold Pathology (LFP) score in association with core clinical features
PD: Parkinson's disease, DLB: dementia with Lewy bodies, MDS-UPDRS III: Movement Disorders Society Unified Parkinson's Disease Rating Scale Part III, MoCA: Montreal Cognitive Assessment
Spearman (Hoehn and Yahr) and Pearson (MDS-UPDRS III and MoCA) correlation coefficients as well as *p*-values corrected for age and sex are shown for each single association.

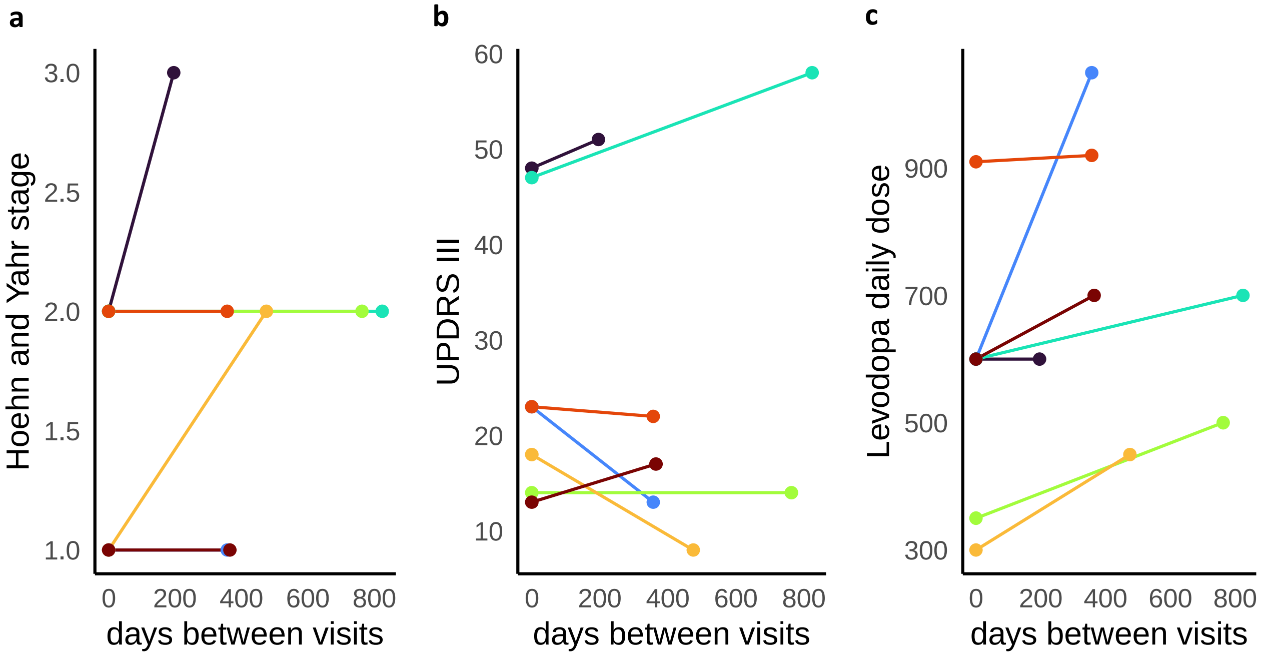

Supplementary Figure S7. Longitudinal investigation of Lewy-Fold Pathology (LFP) score in 7 individuals with PD

PD: Parkinson's disease, MoCA: Montreal Cognitive Assessment

LFP scores were determined of CSF of two serial lumbar punctures from 7 PD patients. LFP scores from the same patient are connected by a line. Each patient is shown in a specific color along **a)** – **c)** and associations with clinical measures of disease severity are presented. In **a)** the change of the Hoehn and Yahr stage over time is depicted, **b)** shows the corresponding change in MDS-UPDRS III and **c)** the change of Levodopa daily dose over time. Arrows connect baseline with follow-up values of each individual patient.

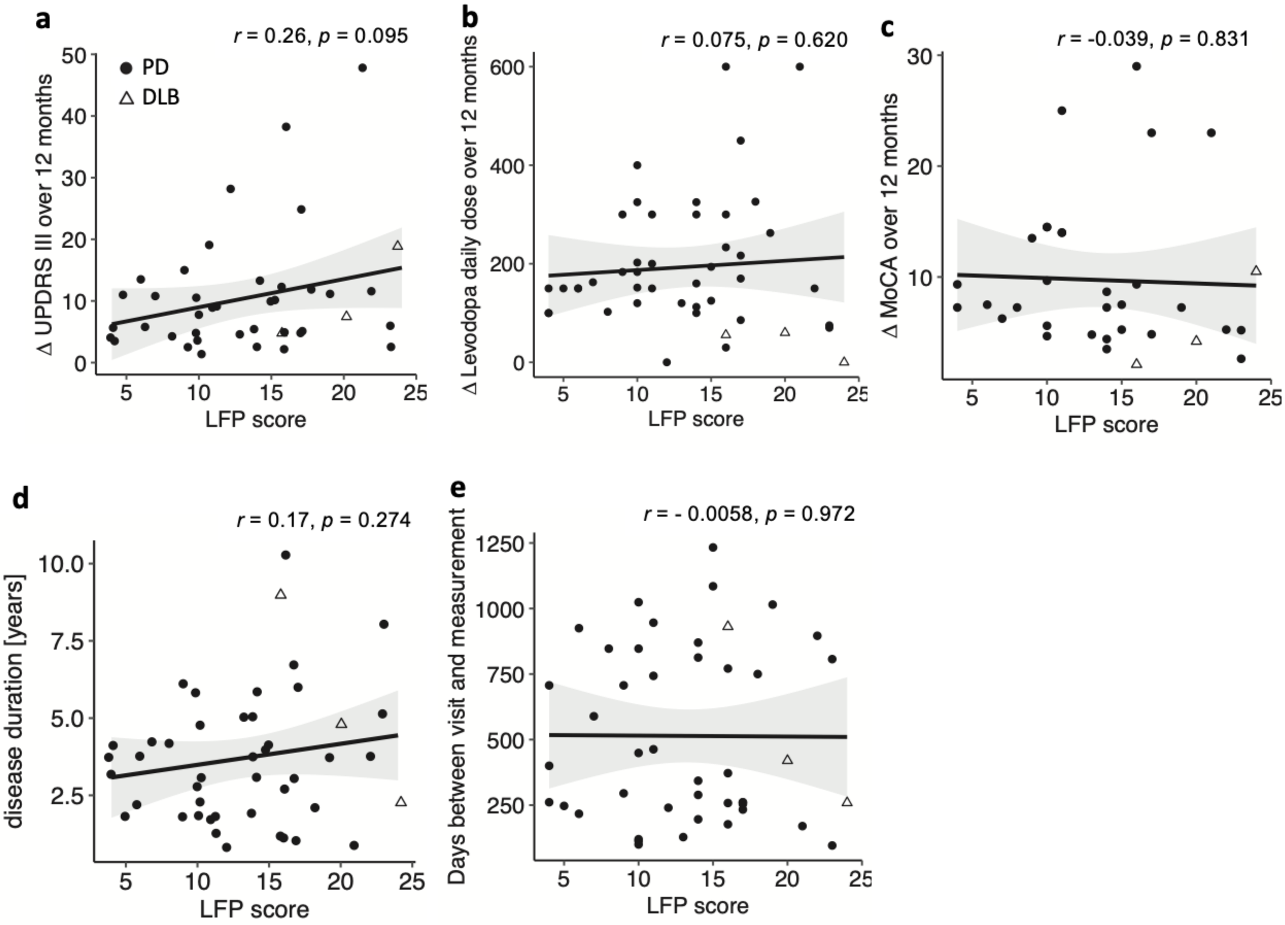

Supplementary Figure S8. Lewy-Fold Pathology (LFP) score in association with longitudinal change of disease severity scores and storage time

DLB: dementia with Lewy bodies, PD: Parkinson's disease, UPDRS III: Movement Disorders Society Unified Parkinson's Disease Rating Scale Part III, MoCA: Montreal Cognitive Assessment

In **a)** – **c)** bivariate associations between the change of disease severity scores over 12 months UPDRS III (**a**), Levodopa daily dose (**b**) MoCA (**c**) disease duration (**d**) and the Lewy fold pathology score (LFP score) are presented. In **e**) the bivariate association between the storage time of CSF at -80°C until measurement and the LFP score is visualized. The LFP score shows does not show any significant associations with the depictecd variables. Pearson coefficients as well as *p*-values corrected for age and sex are shown for each single association, regression lines (black) and 95% confidence intervals (grey) are provided for continuous variables. For discrimination of diagnoses PD and DLB samples are presented by dots and triangles, respectively.

Supplementary Table 5. Literature overview on aSyn SAAs in CSF of PD, DLB, iRBD patients, clinical correlates and quantification approaches

Significant/meaningful correlations appear in bold black, not significant/not meaningful correlations appear in bold red.

- Data from the Soto laboratory and Amprion: Shahnawaz et al. utilized T_{50} as the primary parameter and reported significant negative correlations with H&Y stages in both Japanese and German cohorts (r = -0.54, p = 0.006 and r = -0.36, p = 0.02, respectively). Concha-Marambio et al. explored several kinetic parameters, including F_{max}, T_{50}, slope and TTT. In PD cases, no significant correlations were found between these parameters and clinical measures such as MDS-UPDRS III and MoCA. In contrast, in patients with iRBD, mean and median F_{max} negatively correlated with total MDS-UPDRS scores (r = -0.54, -0.57) and p-tau/t-tau ratio (r = -0.73, -0.82). Additionally, T_{50} and TTT positively correlated with the CSF/serum albumin ratio (r = 0.67, -0.69). Longitudinal changes in MDS-UPDRS III were negatively associated with $\log_{10}$(mean F_{max}) after adjusting for sex, age and diagnosis group, indicating that higher F_{max} corresponds to worse disease status (p = 0.047). Eijsvogel et al.[48] demonstrated that reduction in F_{max} was more pronounced and statistically significant in individuals with detectable CSF antibody titers, suggesting a potential route to quantifiable assessment of treatment effects on αSyn pathology in clinical trials.
- Data from the Green laboratory: Poggiolini et al. found that while there was some evidence of association between T_{50} and clinical parameters such as MDS-UPDRS I and MoCA, the direction of these associations was contrary to expectations. Additionally, there was weak evidence for the association of worse MDS-UPDRS IV scores with higher F_{max} values, which became less significant after adjusting for age, disease duration and sex.
- Data using the assay from the Caughey laboratory: Orru et al. reported no significant correlations between T_{50} values and clinical measures of MDS-UPDRS III, MDS-UPDRS total and MoCA scores for PD patients. Brockmann et al. reported that higher AUC was associated with higher MDS-UPDRS III scores (r = 0.150, p = 0.034) and lower MoCA scores (r = -0.189, p = 0.009). Shorter TTT was associated with higher MDS-UPDRS III (r = -0.165, p = 0.020) and lower MoCA scores (r = 0.186, p = 0.011). Bräuer et al. examined TTT2 and TTT. TTT2 and TTT were positively correlated with MoCA scores across the entire cohort and within PD and DLB subpopulations. TTT2 also showed a moderate negative correlation with MDS-UPDRS III scores, suggesting that shorter TTT2 is associated with more severe motor symptoms. Brockmann et al. found that more positive replicates, higher Fmax, AUC and shorter TTT were associated with both the presence of cognitive impairment and lower MoCA scores.
- Comparative studies: of note, 2 publications compared different SAAs in the same study: Russo et al. analyzed F_{max}, AUC, TTT, T_{50} and endpoint dilution (SD_{50}) across three different laboratories (AbbVie, Amprion and Caughey). AbbVie focused on the kinetic parameters Fmax, AUC and TTT. Significant correlations were found between these parameters and University of Pennsylvania Smell Identification Test (UPSIT) and MDS-UPDRS I scores. However, no consistent correlations with other clinical measures like MDS-UPDRS III or MoCA were observed. Amprion also analyzed F_{max}, AUC and TTT, finding significant correlations with UPSIT scores. However, similar to the findings at Abbvie, no consistent correlations were observed with broader clinical measures. The Caughey laboratory took a more comprehensive approach, including SD_{50} to quantify relative amounts of seeding activity. They found positive correlations between SD_{50} and age (r = +0.36, p = 0.006), disease duration (r = +0.31, p = 0.02) and NfL levels (r = +0.51, p = 0.05). Despite these findings, no consistent correlations were observed between SD_{50} and clinical measures such as MDS-UPDRS III or MoCA. Kang et al. focused on T_{50} across two different laboratories (Soto, Green). The Soto laboratory evaluated T_{50} values in a cohort of 100 PD patients. The study found no significant correlations between T_{50} values and disease characteristics such as H&Y stage (R^2 = 0.0099, p = 0.3235), MDS-UPDRS III (R^2 = 0.0013, p = 0.7202) and MDS-UPDRS total scores (R^2 = 0.0004, p = 0.8458). The Green laboratory also evaluated T_{50} values in a cohort of 101 PD patients. Similar to the findings from the Soto laboratory, no significant correlations were found between T_{50} values and clinical measures including H&Y stage (R^2 = 0.0093, p = 0.3365), MDS-UPDRS III (R^2 = 0.0039, p = 0.5338) and MDS-UPDRS total scores (R^2 = 0.0100, p = 0.3204).

Study	Year	Quanti-fication assessed	Kinetic parameters	Clinical parameters with correlations	Method
Shahnawaz et al., JAMA Neurolology	2017	yes	T_{50}	PD cases (n=76),<br>**Significant correlation between T_{50} and Hoehn and Yahr stages:**<br>- **Japanese cohort: Spearman r -0.54, p 0.006**<br>- **German cohort: Spearman r -0.36, p 0.02**	Soto
Kang et al., Movement Disorders	2019	yes	T_{50}	PD cases (n=100 Soto, n=101 Green),<br>**No significant correlations were observed between T_{50} values and disease characteristics (H&Y stage, MDS-UPDRS III, MDS-UPDRS total scores) for PD patients**<br>• **Soto (n = 100): T_{50} vs. H & Y stage: R^2 = 0.0099, P = 0.3235 (not significant), T_{50} vs. MDS-UPDRS III: R^2 = 0.0013, p = 0.7202, T_{50} vs. UPDRS total scores: R^2 = 0.0004, p = 0.8458 Allison Green (n = 101): T_{50} vs. H&Y stage: R^2 = 0.0093, p = 0.3365, T_{50} vs. MDS-UPDRS III: R^2 = 0.0039, p = 0.5338, T_{50} vs. MDS-UPDRS total scores: R^2 = 0.0100, p = 0.3204**	Soto, Green
Orru et al., Annals of Clinical and Translational Neurology	2020	yes	T_{50}	PD cases (n=108),<br>**No significant correlations of T50 with MDS-UPDRS III, MDS-UPDRS total, MoCA**	Caughey
Russo et al. Acta Neuropathol Commun	2021	yes	F_{max}, AUC, TTT, T_{50}, SD_{50} (derived from endpoint dilution)	PD cases (n=30 PD BL & Y3 - 60 samples),<br>• **SD_{50} (Caughey only): positive correlation with age (r = 0.36, p = 0.006), positive correlation with disease duration (r = +0.31, p = 0.02), positive correlation with NfL (r = +0.51, p = 0.05, no significant correlations with MDS-UPDRS III or MoCA**<br>• **F_{max}, AUC, TTT (AbbVie): significant correlations with UPSIT and MDS-UPDRS I, no significant correlations with MoCA or MDS-UPDRS III**<br>• **F_{max} (Amprion): significant correlation with UPSIT, no significant correlations with MoCA or MDS-UPDRS III**<br>• **F_{max}, AUC, TTT (Caughey): significant correlations with MDS-UPDRS II, III, MDS-UPDRS total, and disease duration, no significant correlations with MoCA**<br>• **Paired baseline to year 3 samples (longitudinal) – (Caughey only): no consistent signal changes at the single individual level**	Amprion, AbbVie, Caughey
Brockmann et al., Acta Neuropathol Commun	2021	yes	AUC, F_{max}, TTT	PD cases (n=236) and DLB cases (n=49),<br>• **Higher AUC associated with: higher MDS-UPDRS III (r = 0.150, p = 0.034), lower MoCA scores (r = -0.189, p = 0.009)**<br>• **Shorter TTT associated with: higher MDS-UPDRS III (r = -0.165, p = 0.020), lower MoCA scores (r = 0.186, p = 0.011)**	Caughey
Poggiolini et al., Brain	2022	yes	F_{max}, AUC, TTT, T_{50}	PD cases (n=74) and iRBD cases (n=45),<br>• **T_{50} and MDS-UPDRS I: p = 0.003 (not in expected direction, i.e., shorter T_{50} with more severe phenotype), MoCA: p = 0.04 (not in expected direction)**<br>• **Fmax and MDS-UPDRS IV: p = 0.05 (weak evidence, association attenuated after adjustment for age, disease duration, and sex)**	Green

Study	Year	Quanti-fication assessed	Kinetic parameters	Clinical parameters with correlations	Method
Concha-Marambio et al., Movement Disorders	2023	yes	F_{max}, T_{50}, Slope, TTT	PD cases (n=113):<br>• **No relevant correlations between MDS-UPDRS III, MoCA, MMSE mean and median**<br>iRBD cases (n=29):<br>• **Mean and median F_{max} negatively correlated with: Total MDS-UPDRS (r = -0.54, -0.57), p-tau/t-tau ratio (r = -0.73, -0.82),**<br>• **T_{50} and TTT (both mean and median) positively correlated with: CSF/serum albumin ratio (r = 0.69, 0.67, 0.68, 0.69),**<br>• **Log_{10}(mean F_{max}) at baseline and longitudinal changes in MDS-UPDRS III showed negative association after adjusting for sex, age, and diagnosis group (p = 0.047)**<br>• **Log_{10}(median F_{max}) at baseline and MDS-UPDRS III, MDS-UPDRS total, NMS sum, and GDS sum scores showed negative association with after adjusting for sex, age, and diagnosis group (p = 0.045, p = 0.028, p = 0.012, and p = 0.043, respectively)**<br>• **MoCA total score showed significant positive associations with the log10(mean slope) (p = 0.007) and log10(median slope) (p = 0.011) at BL**	Amprion
Bräuer et al., Acta Neuropathol Commun	2023	yes	TTT2, TTT	PD cases (n=28) and DLB cases (n=47),<br>• **TTT2 and MoCA: entire cohort: Spearman's rho = 0.519, 95% CI = 0.297–0.688, p < 0.0001, PD patients: Spearman r= 0.550, 95% CI = 0.153–0.793, p = 0.008, DLB patients: Spearman r = 0.335, 95% CI = 0.003–0.601, p = 0.043**<br>• **TTT and MoCA: entire cohort: Spearman r= 0.412, 95% CI = 0.167–0.609, p < 0.01, PD patients: Spearman r = 0.500, 95% CI = 0.087–0.767, p = 0.018, DLB patients: Spearman r = 0.349, 95% CI = 0.018–0.611, p = 0.034**<br>• **TTT2 and MDS-UPDRS III: negative correlation: Spearman's rho = -0.469, 95% CI = -0.694 to -0.161, p < 0.01**<br>• **Linear models with TTT2: MoCA and MDS-UPDRS III were significant factors in predicting TTT2 when included individually: MoCA: ANOVA, F = 13.25, p = 0.001, MDS-UPDRS III: ANOVA, F = 10.04, p = 0.003, MoCA remained significant when added after MDS-UPDR -III (ANOVA, F = 5.76, p = 0.023), MDS-UPDRS III was not significant when added after MoCA (ANOVA, F = 2.56, p = 0.120)**	Caughey
Brockmann et al., NPJ Parkinson's Disease	2024	yes	F_{max}, AUC, TTT, Number of positive replicates	PD cases (n=199),<br>• **Higher number of positive seeding replicates and presence of cognitive impairment: r = 0.215, p = 0.003**<br>• **Shorter TTT phase and presence of cognitive impairment: r = -0.221, p = 0.002**<br>• **Higher F_{max} and presence of cognitive impairment: r = 0.186, p = 0.009**<br>• **Higher AUC and presence of cognitive impairment: r = 0.209, p = 0.003**<br>• **Higher number of positive seeding replicates and MoCA: r = -0.346, p ≤ 0.001**	Caughey
Eijsvogel et al., Nature Medicine	2024	yes	F_{max}	PD cases (n=20),<br>**Reduction of F_{max} was more pronounced and statistically significant in individuals with detectable CSF antibody titers (time effect: F = 12.77, p = 0.0002; treatment × time effect: F = 6.755, p = 0.0037)**<br>Correlations with MoCA or MDS-UPDRS III not shown	Soto

Study	Year	Quanti-fication assessed	Kinetic parameters	Clinical parameters with correlations	Method
Srivastava et al., Plos Pathogens	2024	yes	2F8R vs. 10F4R:<br>• 2F8R: 2-fold serial dilutions with 8 replicates per dilution. This format increased precision and reduced variability in detecting smaller differences in seed concentrations.<br>• 10F4R: 10-fold dilutions with 4 replicates per dilution. Less sensitive and more prone to variability.<br><br>midSIN Algorithm: Used Bayesian inference for accurate estimation of SD50 (concentration at which 50% of wells are positive). More robust than Spearman-Kärber or Reed-Muench methods.	PD and DLB cases:<br>• Improved precision in distinguishing 2-fold differences in seed concentrations using midSIN and Poisson methods in endpoint dilution (ED) SAA.<br>• Validation across multiple biospecimens (brain tissue, CSF, skin, and OM).<br>• No explicit clinical correlation tested, but assay demonstrated high reproducibility and quantitative accuracy, which supports clinical applicability in future studies.	Caughey
Fairfoul et al., Annals of Clinical and Translational Neurology	2016	no			
Groveman et al., Acta Neuropathol Commun	2018	no			
Bongianni et al., Annals of Clinical and Translational Neurology	2019	no			
De Luca et al., Translational Neurodegeneration	2019	no			
van Rumund et al., Annals of Neurology	2019	no			
Rossi et al., Acta Neuriopathologica	2020	no			
Shahnawaz et al., Nature	2020	no			
Singer et al., Annals Neurology	2020	no			
Bargar et al., Acta Neuropathol Commun	2021	no			
Iranzo et al., Lancet Neurology	2021	no			
Quadalti et al., NPJ Parkinson's Disease	2021	no			
Rossi et al., Neurology	2021	no			

Study	Year	Quanti-fication assessed	Kinetic parameters	Clinical parameters with correlations	Method
Sokratian et al., Acta Neuropathologica	2021	no			
Arnold et al., Annals of Neurology	2022	no			
Garrido et al., Movement Disorders	2022	no			
Martinez-Valbuena et al., Acta Neuropathologica	2022	no			
Fernandes Gomes et al., Parkinsonism and Related Disorders	2023	no			
Iranzo et al., Neurology	2023	no			
Middleton al., Journal of Neurology	2023	no			
Siderowf et al., Lancet Neurology	2023	no			
Bentivenga et al., Acta Neuropathologica	2024	no			
Mammana et al., Clin Chem Lab Med	2024	no			
